# Cellular senescence in hepatocellular carcinoma induced by a long non-coding RNA-encoded peptide PINT87aa by blocking FOXM1-mediated *PHB2*

**DOI:** 10.7150/thno.55672

**Published:** 2021-03-04

**Authors:** Xiaohong Xiang, Yunong Fu, Kun Zhao, Runchen Miao, Xing Zhang, Xiaohua Ma, Chang Liu, Nu Zhang, Kai Qu

**Affiliations:** 1 Department of Hepatobiliary Surgery, The First Affiliated Hospital of Xi'an Jiaotong University, Xi'an, Shaanxi, 710061, PR China.; 2 Department of Neurosurgery, The First Affiliated Hospital of Sun Yat-sen University, Guangzhou, Guangdong, 510080, PR China.

**Keywords:** hepatocellular carcinoma, PINT87aa, cellular senescence, FOXM1, mitophagy.

## Abstract

**Rationale:** Recently, long non-coding RNAs (lncRNAs), known to be involved in human cancer progression, have been shown to encode peptides with biological functions, but the role of lncRNA-encoded peptides in cellular senescence is largely unexplored. We previously reported the tumor-suppressive role of PINT87aa, a peptide encoded by the long intergenic non-protein coding RNA, p53 induced transcript (*LINC-PINT).* Here, we investigated PINT87aa's role in hepatocellular carcinoma (HCC) cellular senescence.

**Methods:** We examined PINT87aa and truncated PINT87aa functions *in vitro* by monitoring cell proliferation and performed flow cytometry, senescence-associated β-galactosidase staining, JC-1 staining indicative of mitochondrial membrane potential, the ratio of the overlapping area of light chain 3 beta (LC3B) and mitochondrial probes and the ratio of lysosomal associated membrane protein 1 (LAMP1) overlapping with cytochrome c oxidase subunit 4I1 (COXIV) denoting mitophagy. PINT87aa and truncated PINT87aa functions *in vivo* were verified by subcutaneously transplanted tumors in nude mice. The possible binding between PINT87aa and forkhead box M1 (FOXM1) was predicted through structural analysis and verified by co-immunoprecipitation and immunofluorescence co-localization. Rescue experiments were performed *in vivo and in vitro* following FOXM1 overexpression. Further, chromatin immunoprecipitation, polymerase chain reaction, and dual-luciferase reporter gene assay were conducted to validate FOXM1 binding to the *prohibitin 2* (*PHB2*) promoter.

**Results:** PINT87aa was significantly increased in the hydrogen peroxide-induced HCC cell senescence model. Overexpression of PINT87aa induced growth inhibition, cellular senescence, and decreased mitophagy *in vitro* and *in vivo*. In contrast, FOXM1 gain-of-function could partially reduce the proportion of senescent HCC cells and enhance mitophagy. PINT87aa overexpression did not affect the expression of FOXM1 itself but reduced that of its target genes involved in cell cycle and proliferation, especially *PHB2,* which was involved in mitophagy and transcribed by FOXM1. Structural analysis indicated that PINT87aa could bind to the DNA-binding domain of FOXM1, which was confirmed by co-immunoprecipitation and immunofluorescence co-localization. Furthermore, we demonstrated that the 2 to 39 amino acid truncated form of the peptide exerted effects similarly to the full form.

**Conclusion:** Our study established the role of PINT87aa as a novel biomarker and a key regulator of cellular senescence in HCC and identified PINT87aa as a potential therapeutic target for HCC.

## Introduction

Cellular senescence is an anti-tumor program, which induces proliferation arrest in tumor cells 1. In general, there are two types of senescence, replicative senescence (RS) caused by telomere shortening or dysfunction 2, 3 and premature senescence triggered by stress, including oncogenes (oncogene-induced senescence, OIS) 4, 5 and DNA damage response (DDR) 6. Replicative and premature senescence exhibit similar phenotypes and molecular characteristics 7. Over the past decade, accumulating evidence indicated that inducing cancer cells into senescence could be a potential anti-cancer therapy 8. In hepatocellular carcinoma (HCC), cellular senescence was shown to be mainly controlled by p53-dependent or -independent signaling 6, 9. Further, cirrhotic liver tissue containing a higher proportion of senescent liver cells could undergo malignant transformation after breaking through the senescence barrier 10. Therefore, exploring the diversity of senescence-associated mechanisms in HCC would be helpful for HCC treatment.

Over the past few years, long non-coding RNAs (lncRNAs), have attracted increasing interest. LncRNAs play crucial roles in various diseases, especially contributing to the abnormality of gene products involved in the progression of human cancers 11, 12. It has also been demonstrated that lncRNAs could serve as prognostic or diagnostic indicators due to their extensive involvement in gene regulation networks 13-15. Recently, several lncRNAs have been identified that encode peptides with biological functions as another mode of lncRNA-mediated effects 16, 17. Although several peptides encoded by lncRNAs have been identified, only a handful have been functionally characterized in cellular senescence, and many of them require further verification. Previously, we first reported that the long intergenic non-protein coding RNA, p53-induced transcript (LINC-PINT) played a tumor suppressor role in early-stage glioma. Its exon 2 could be looped and translated into a peptide consisting of 87 amino acids, and the LINC-PINT function was carried out mainly through the peptide product, PINT87aa 18. Herein, we investigated the effect and underlying molecular mechanism of PINT87aa in HCC. We identified the functional fragment of PINT87aa by truncating the peptide. Our data shed light on the underlying function of PINT87aa encoded by *LINC-PINT* in mediating cell cycle arrest, cellular senescence, and mitophagy in HCC cells, thus providing evidence for its potential as a therapeutic target in HCC.

## Materials and Methods

### Cell culture and tissue collection

Human HCC cell lines, Bel7404, HHCC, MHCC-97H, Huh-7, Hep3B, SMMC-7721, and HepG2, were purchased from the Stem Cell Bank, Chinese Academy of Sciences, and cultured in RPMI-1640 or DMEM with 10% fetal bovine serum. Senescent primary hepatocytes (SPHC) were extracted from human liver tissues. Samples from 8 HCC patients with moderate or severe liver cirrhosis were collected at the first affiliated hospital of Xi'an Jiaotong University. The study was approved by the Ethics Committee of Xi'an Jiaotong University. All participants in this study provided written informed consent.

### Vectors and transfection

Recombinant expression vectors of LINC-PINT (NR_109851), PINT87aa, truncated PINT87aa-GFP (lacking 30 to 36 amino acids), FOXM1, and PHB2 were provided by GeneChem (Shanghai, China). HCC cells overexpressing PINT87aa, FOXM1, and PHB2 were generated through stable transfection of an adenovirus vector system. Truncated forms of PINT87aa were synthesized by Qiangyao Biological Technology (Suzhou, China).

### Cell proliferation analysis

For colony formation assays, fixed cells were stained with 0.1% crystal violet after 2 weeks of incubation. Colonies that contained over 50 cells were included in the calculation range.

For EdU assays, cells were seeded in a 96-well plate. Thereafter, EdU labeling, Apollo, and DNA staining were performed according to the manufacturer's instructions using the Kit obtained from Ruibo (Guangzhou, China). The number of Edu-positive cells was counted after image capturing (Zeiss 2.0, Germany).

### Cell cycle analysis

Collected cells were fixed overnight at 4 °C in 70% ethanol. The liquid was discarded after centrifugation, and 0.5 ml of staining solution was added to every sample according to the manufacturer's instructions (Qihai, Shanghai, China). Samples were then incubated at 37 °C for 30 min, and the cells were measured by flow cytometry (BD, USA).

### Real-time PCR (qRT-PCR)

Total RNA was extracted by TRIzol reagent (Ambion) and was determined by Qubit fluorometer (Thermo Fisher). The SYBR® Kit (TaKaRa) was used to perform qRT-PCR according to the manufacturer's instructions. All genes were normalized to the expression level of β-actin. Relative expression was calculated using the comparative Ct method (ΔΔCt method). Primer sequences are shown in Table S1.

### Immunohistochemistry staining (IHC) and Western blotting

The expression of PINT87aa, FOXM1, PHB2, and Ki67 in tumor tissues was examined by fixing tissue slides with 4% paraformaldehyde. The slides were blocked with goat serum, incubated with primary antibodies, followed by secondary antibodies (SP9001, Zhongshan Jinqiao Biotechnology, Beijing, China) and diaminobenzidine tetrahydrochloride (DAB) (Zhongshan Jinqiao Biotechnology, Beijing, China). The slides were observed under a microscope (Zeiss 2.0, Germany). The details of primary antibodies were listed in Table S2.

For Western blotting, RIPA lysis buffer was used to extract total proteins and the Mitochondria Isolation Kit (Beyotime, Shanghai, China) was used to obtain mitochondrial proteins. Qubit fluorometer (Thermo Fisher) was used to determine protein concentration. The protein concentrations of 50 μg PINT87aa and 10 μg of other protein samples were separated by 10%, 12%, 15% and tricine SDS-PAGE and transferred to nitrocellulose membranes (Millipore, Merck, Germany). After incubating with primary antibodies at 4 ºC overnight and secondary antibodies at 37 ℃ for 1 h, protein signals were detected by the ECL detection system (Amersham Imager 600 and BioRad, USA). The details of antibodies used for Western blotting were provided in Table S2.

### SA-β-Gal staining analysis

For SA-β-Gal staining, frozen sections of mouse and human tissues were used. After rewarming, sections were fixed for 15 min, fully covered with staining solution, and incubated at 37 °C overnight. Subsequently, staining-positive cells were counted under a microscope. After transfection or truncated peptide treatment, the same staining process was employed using the staining kit from Beyotime (Shanghai, China).

For fluorometric SA-β-Gal staining (Cell Biolabs, USA), cells were incubated at 37ºC for 2 h in 2 ml of Cell Pretreatment Solution. Subsequently, 10 µl of 200 X SA-ß-Gal substrate solution was added and incubated at 37 ºC for 4 h to overnight. After trypsinization and washing, cells were analyzed by flow cytometry (BD, USA).

### RNA fluorescence *in situ* hybridization (FISH)

The labeled PINT87aa fluorescent probe was synthesized by GenePharma. Cells were fixed on the slide, denatured at 65 °C, incubated with 25 µg/ml probe, washed, stained with 4'-6-diamidino-2-phenylindole (DAPI), and images were captured by Leica confocal microscope. The kit was purchased from GenePharma (Shanghai, China), and all procedures were performed following the manufacturer's instructions.

### Immunofluorescence staining

Cells were seeded in a confocal dish, fixed with 4% paraformaldehyde, treated with 1 x PBS containing 0.1% Triton X-100 for 2 min, and blocked with 1% goat serum at room temperature for 30 min. After incubating with the appropriate primary antibody at 4 °C overnight and secondary antibody for 1 h, images were captured using a Leica confocal microscope. The details of antibodies used for immunofluorescence staining were in Table S2.

### Dual-luciferase reporter gene assay

Cells were inoculated in a 96-well plate and cell lysis buffer was transferred to the black microplate, then firefly luciferase reaction solution was added and the firefly luciferase activity was determined. After inocubating with Renilla luciferase reaction solution, the activity was measured. The kits used were obtained from Yeasen (China).

### Chromatin immunoprecipitation (ChIP)-qPCR

ChIP-qPCR was employed to analyze genomic DNA sequences bound to the FOXM1 protein (CST) using NovoNGS^®^ CUT&Tag 2.0 ChIP kits (Novoprotein, Shanghai, China). Briefly, protein-DNA complexes were crosslinked, immunoprecipitated, purified, and then amplified for the *PHB2* promoter sequence using qPCR.

### Co-immunoprecipitation (Co-IP)

We incubated cell lysates with antibodies for 2 h and added protein A/G magnetic beads to the antigen/antibody complex for 1 h at room temperature. Then the beads were washed twice with immunoprecipitation lysis/washing buffer and antigen/antibody complexes eluted. SDS-PAGE electrophoresis was performed. The kit used was obtained from Thermo Fisher (MA, USA).

### Mitochondrial imaging by confocal microscopy

The cells were seeded in a confocal dish and transfected with the GFP-LC3B vector. After discarding the medium, cells were incubated with the fluorescent dye Mito-Tracker Red (Beyotime, Shanghai, China) diluted 1:10000 for 30 min at 37 ℃. Next, the medium was changed with the pre-warmed medium at 37 ℃. Finally, the mitophagic activity was determined from the average number of mitochondrial GFP-LC3B from 5 high-power fields using Leica confocal microscopy.

### Mitochondrial membrane potential (MMP) detection

After HCC cells (2 × 10^4^) were transfected or treated in a 6-well plate, JC-1 dye was added for 20 min at 37 °C. The cells were washed twice and the images were captured under a fluorescence microscope (Zeiss 2.0, Germany). The kit used was obtained from Beyotime (Shanghai, China).

### Subcutaneous xenograft assay

Athymic nude mice were obtained from the Laboratory of Animal Breeding and Research Center, Xi'an Jiaotong University. The xenograft tumor model was established in mice by subcutaneously inoculating 1 x 10^7^ MHCC-97H cells stably expressing a red fluorophore. Tumor development was evaluated three weeks after inoculation. The protocol of animal experiments was approved by the Institutional Animal Care and Use Committee of Xi'an Jiaotong University.

### Prediction of FOXM1 binding site on PINT87aa

We used PSI-PRED to predict the secondary structure of PINT87aa and determined the FOXM1 binding site on PINT87aa 19. The protein structure file of FOXM1 was obtained from the PDB database (http://www.rcsb.org/). We then used AutoDock for molecular docking to select the lowest binding energy conformation.

### Statistical analysis

Statistical analyses were performed using the SPSS software (Version 24). The Student's t-test was used to analyze the differences between continuous variables. A *P*-value lower than 0.05 indicated statistical significance.

Other methods involved in this study were listed in supplementary materials.

## Results

### PINT87aa was overexpressed in senescent HCC cells

We first used an *in silico* senescence model to identify senescence-associated lncRNAs (SA-lncRNAs), for which the workflow is shown in Figure S1A. The comparative analysis detected 4 upregulated and 2 downregulated SA-lncRNAs (Figure S1B). To validate the candidate SA-lncRNAs in senescent HCC cells, we first determined the efficiency of hydrogen peroxide (H_2_O_2_)-induced senescence in SMMC-7721 and HepG2 (wild-type *TP53*) cells. Our data showed that 100 μM H_2_O_2_ treatment for 24 h could induce cellular senescence in approximately 60-75% HCC cells (Figure S1C-D). Therefore, we selected 100 μM H_2_O_2_ for 24 h as the treatment to establish senescent HCC cell models. The model was also validated in MHCC-97H cells (Figure S1E). We then detected the expression of 6 differentially expressed candidate SA-lncRNAs in H_2_O_2_-treated HCC cells. Among them, LINC-PINT was significantly and differentially expressed in senescent cells (Table S3), suggestive of its involvement in cellular senescence of HCC.

**Figure 1:**
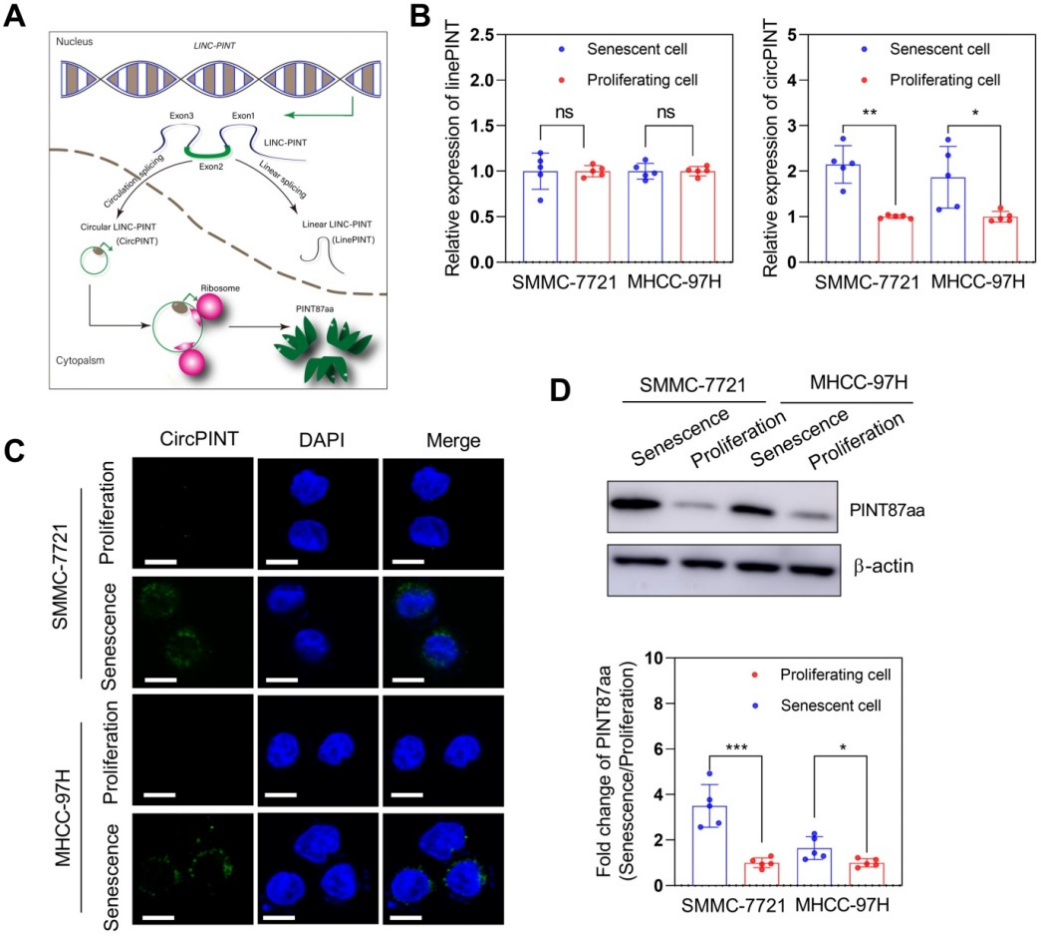
** PINT87aa was overexpressed in H_2_O_2_-induced senescent HCC cells.** (A) Schematic diagram of NR_109851, transcription of LINC-PINT generating linear PINT (linePNT) and circular PINT (circPINT) that could encode the PINT87aa peptide. (B) qRT-PCR detected circPINT and linePINT expression in senescent and proliferating SMMC-7721 and MHCC-97H cells. (C) FISH detected circPINT expression in senescent and proliferating SMMC-7721 and MHCC-97H cells. Scale bars = 15 μm. (D) Western blotting detected PINT87aa expression in senescent and proliferating SMMC-7721 and MHCC-97H cells. *P < 0.05; **P < 0.01; ***P < 0.001; ns *P* > 0.05.

We examined the circPINT level in senescent primary hepatocytes (SPHC) and HCC cell lines to explore whether LINC-PINT encoded peptide, PINT87aa, plays a role in senescent cells. The results revealed that circPINT expression in SPHC was much higher than in seven HCC cell lines (Figure S2A). Also, expression results for PINT87aa and circPINT were consistent (Figure S2B). Based on these results, we selected SMMC-7721 (wild-type *TP53*) and MHCC-97H (mutant *TP53*) for further studies. The expression of circPINT in senescent SMMC-7721 and MHCC-97H was significantly higher than in their proliferating counterparts (Figure 1B). We used FISH to examine circPINT expression in proliferating and senescent SMMC-7721 and MHCC-97H cells and found it to be mainly localized in the cytoplasm and less expressed in proliferating than senescent SMMC-7721 and MHCC-97H cells (Figure 1C). This observation was confirmed by Western blotting of PINT87aa (Figure 1D).

### Overexpression of PINT87aa induced cellular senescence in HCC cells *in vitro* and *in vivo*

**Figure 2:**
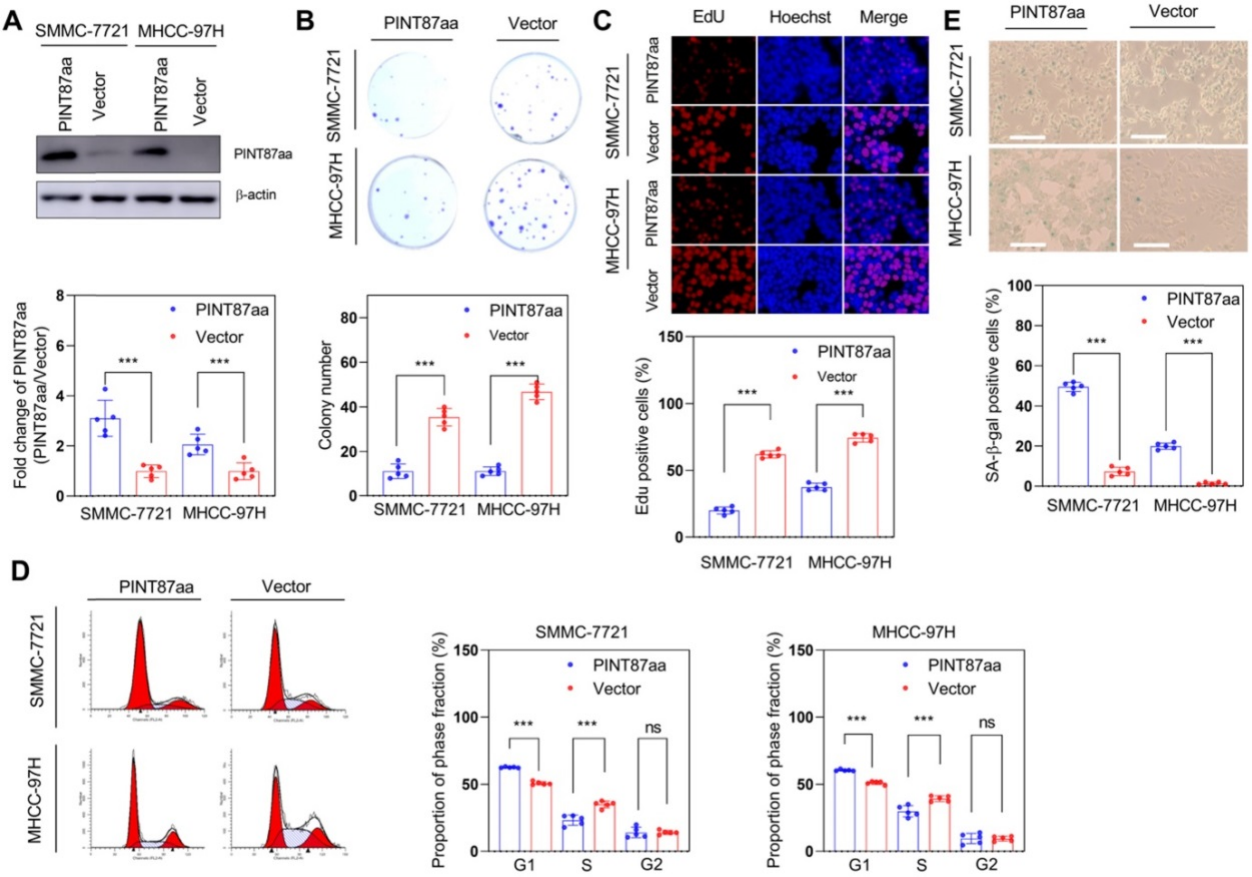
** Overexpression of PINT87aa induced senescence in HCC cells.** (A) Expression of PINT87aa determined in SMMC-7721 and MHCC-97H cells after PINT87aa transfection. (B and C) Cell viabilities of HCC cells with different PINT87aa expression levels detected by colony formation (B) and (C) EdU assay (Magnification is ×100). (D) Cell cycle distribution of HCC cells transfected with PINT87aa overexpression vector and empty vector. (E) SA-β-gal staining in HCC cells with different PINT87aa expression levels (Magnification is ×100). ***P < 0.001; ns *P* > 0.05.

We constructed a circPINT overexpression vector and transfected it into HCC cells to explore the anti-oncogenic role of PINT87aa. After successful transient transfection, PINT87aa was significantly upregulated in HCC cells (Figure 2A). We then compared the proliferative capacity of PINT87aa-overexpressing and empty vector-transfected HCC cells. Our data indicated that PINT87aa overexpression considerably inhibited colony formation and cell proliferation in HCC cells (Figure 2B-C). Cell cycle analysis revealed that, compared to the empty vector group, the proportion of G1 phase cells was increased in PINT87aa-overexpressing cells (Figure 2D). Since cell cycle arrest is considered to be the most important characteristic of cellular senescence, we determined the effect of PINT87aa overexpression on cellular senescence. As expected, the senescent cell proportion was considerably higher in PINT87aa-overexpressing HCC cells than in vector transfected-cells (Figure 2E), which was consistent with CellEvent Senescence Green Flow Cytometry Assay results (Figure S3A).

**Figure 3:**
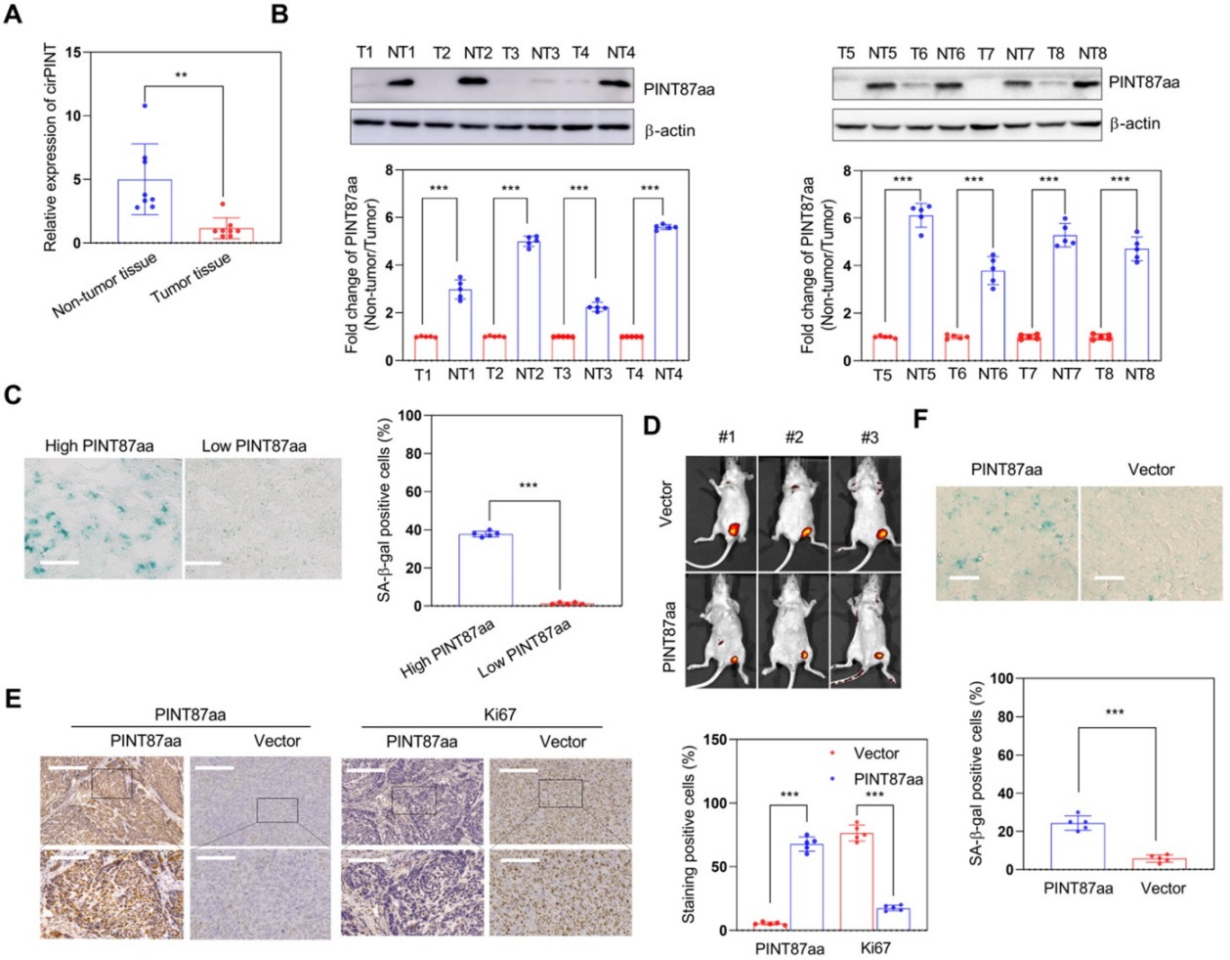
** Overexpression of PINT87aa induced senescence in HCC tissues.** (A and B) Expression of circPINT (n=8) (A) and PINT87aa (n=8) (B) in human HCC (T) and adjacent non-tumor (NT) tissues. (C) SA-β-gal staining in human HCC tissues with different PINT87aa expression levels (Magnification is ×200). (D) Effects of overexpressed PINT87aa on tumor growth by tumorigenicity assay. (E) IHC staining of PINT87aa and proliferative biomarker Ki67 in implanted tumors with different PINT87aa expression levels. The scale bar represents 100μm (top) and 50 μm (bottom). (F) SA-β-gal staining in implanted tumor tissues with different PINT87aa expression levels (Magnification is ×200). **P < 0.01; ***P < 0.001.

Next, we explored the effect of PINT87aa *in vivo* and found the expression of circPINT and PINT87aa in non-tumor tissues to be higher than in adjacent HCC tissues (Figure 3A-B). Also, HCC tissues with high PINT87aa expression exhibited a higher cellular senescence ratio (Figure 3C) and a significant tumor growth delay in the PINT87aa overexpression group (Figure 3D). IHC results confirmed that the PINT87aa overexpression group exhibited higher PINT87aa levels. To explore whether PINT87aa influenced cell proliferation, we performed staining for the proliferation marker Ki67. Our results confirmed that Ki67 was remarkably lower in the PINT87aa overexpression group (Figure 3E). Similarly, SA-β-gal staining in HCC tissues with different PINT87aa expression levels showed a higher proportion of cellular senescence in HCC tissues with increased PINT87aa expression (Figure 3F), suggesting the critical role of PINT87aa in inhibiting cell proliferation and inducing cellular senescence.

### PINT87aa played a pro-senescence role by binding to the DNA-binding domain of FOXM1

**Figure 4:**
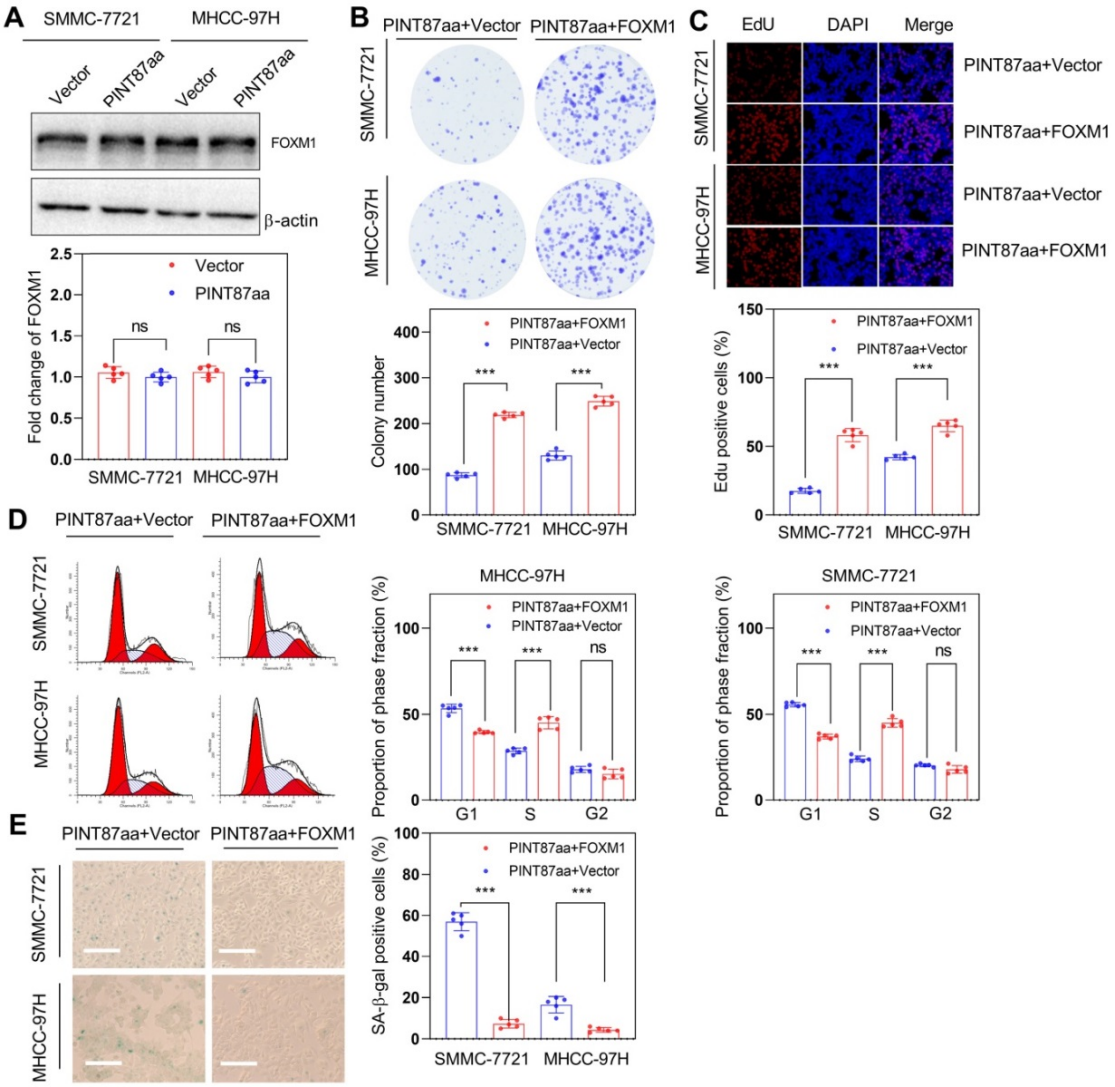
** FOXM1 could reverse the pro-senescence effect of PINT87aa *in vitro*.** (A) Expression of FOXM1 in SMMC-7721 and MHCC-97H cells with stably overexpressed PINT87aa. (B-E) Effects of FOXM1 on cell viabilities (Magnification is ×100) (B and C), cell cycle (D), and cellular senescence (E) (Magnification is ×100) were detected in SMMC-7721 and MHCC-97H cells with stably expressed PINT87aa after FOXM1 transfection. ***P < 0.001; ns *P* > 0.05.

We have previously reported that FOXM1 participates in regulating cellular senescence in HCC 20. Therefore, we examined *FOXM1* expression levels and its target genes involved in cell cycle and proliferation in PINT87aa-overexpressing HCC cells and found no significant change in *FOXM1* mRNA and protein levels under PINT87aa overexpression (Table 1 and Figure 4A). However, the expression levels of its target genes related to cell cycle or proliferation were significantly changed (Table 1), indicating that PINT87aa might play a role by regulating FOXM1 target genes. Therefore, we constructed a *FOXM1* overexpression vector and transfected it into HCC cells stably expressing PINT87aa. Next, we compared the proliferative capacity of FOXM1-overexpressing and empty vector-transfected HCC cells. The data indicated that FOXM1 overexpression significantly restored colony counts (Figure 4B) and cell growth in HCC cells (Figure 4C). Cell cycle analysis showed that compared to the empty vector-transfected cells, the proportion of cells in the G1 phase was significantly lower in FOXM1-overexpressing cells (Figure 4D). Also, the SA-β-gal-positive cells in FOXM1-overexpressing HCC cells were substantially fewer than in the empty vector-transfected cells (Figure 4E), consistent with CellEvent Senescence Green Flow Cytometry Assay results (Figure S3B).

**Figure 5:**
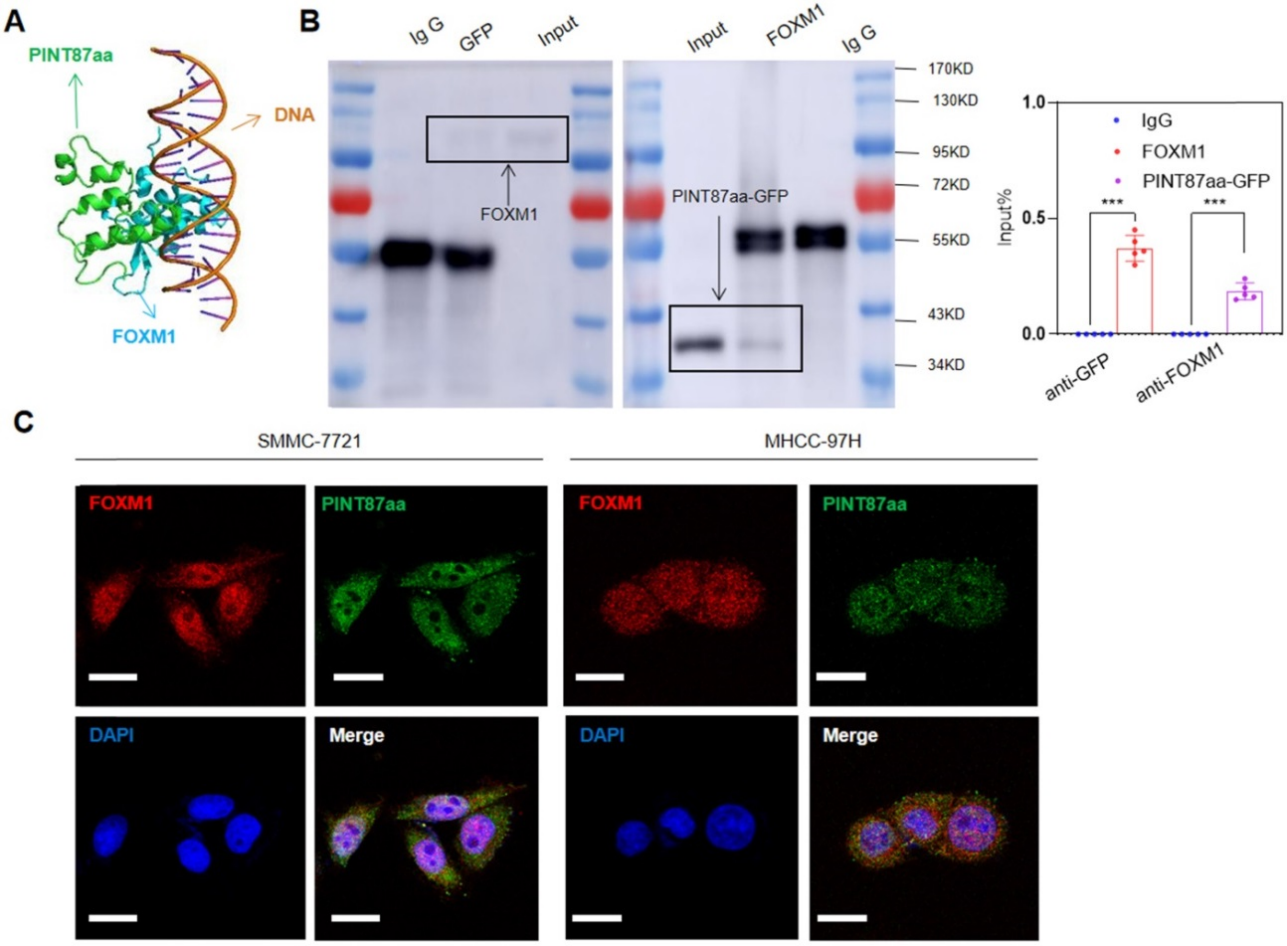
** PINT87aa directly bound to FOXM1.** (A) Schematic diagram of PINT87aa binding to the DNA-binding domain of FOXM1. (B) Co-immunoprecipitation with the anti-GFP antibody in FOXM1 and PINT87aa-GFP co-transfected HEK293 cells and anti-FOXM1 antibody in FOXM1 and PINT87aa-GFP co-transfected HEK293 cells. (C) Immunofluorescent localization of FOXM1 and PINT87aa-GFP in SMMC-7721 and MHCC-97H cells. Scale bars = 20 μm. ***P < 0.001.

We employed binding prediction analysis to investigate how PINT87aa promoted cellular senescence by regulating FOXM1 target genes. As shown in Figure 5A, PINT87aa could bind to the DNA-binding domain of FOXM1, which was confirmed by co-IP results, indicating that PINT87aa-GFP and FOXM1 interacted with each other (Figure 5B). To prevent false-positive results, we conducted a co-IP experiment of GFP and FOXM1 and found no interaction between GFP and FOXM1 (Figure S4A). Finally, we observed co-localization of PINT87aa and FOXM1 through immunofluorescence experiments (Figure 5C). These results indicated the pro-senescence role of PINT87aa by binding to FOXM1 and inhibiting its function.

### PINT87aa blocked FOXM1-mediated transcription of *PHB2*

**Figure 6:**
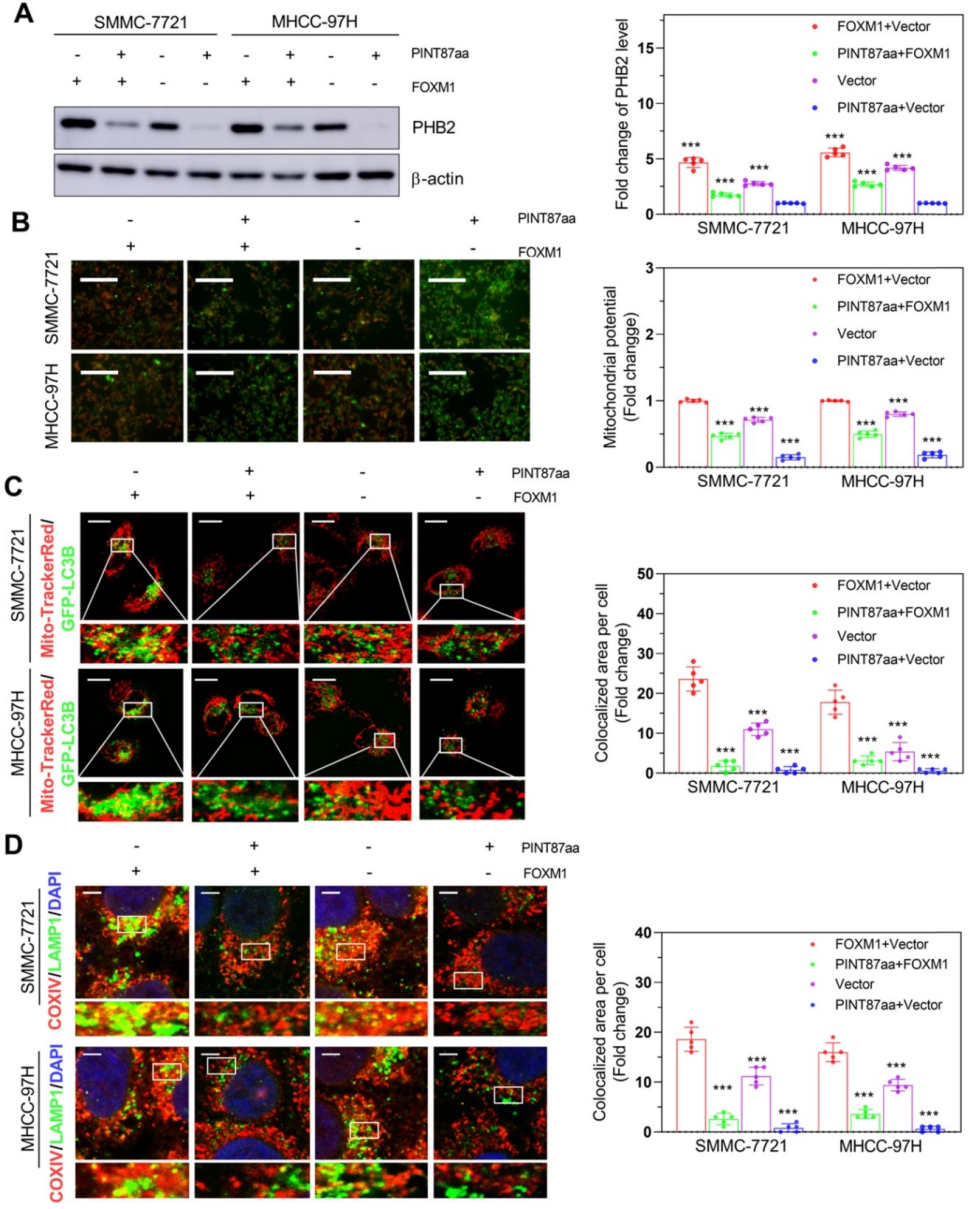
** The effects of PINT87aa and FOXM1 on mitophagy.** (A) Expression of PHB2 in HCC cell lines after transfection with PINT87aa, FOXM1, PINT87aa+FOXM1 or empty vector. (B-D) The change of mitophagy in HCC cell lines after transfection with PINT87aa, FOXM1, PINT87aa+FOXM1 or empty vector: (B) the MMP (Magnification is ×100), (C) the proportion of GFP-LC3B spots overlapping with the mitochondria by MitoTracker Red (Scale bars = 20 μm), (D) and the proportion of LAMP1 indicating lysosome spots overlapping with the mitochondria by COXIV (Scale bars = 5 μm). ***P < 0.001.

Studies have shown differences in mitochondrial functional status between senescent and proliferating cells 21, 22. Therefore, we first acquired FOXM1 target genes through the GEO dataset GSE60032 and selected 82 of these genes related to mitochondrial function (Figure S5A). Among these genes, we found that PHB2 changed remarkably in HCC cells with PINT87aa, FOXM1, or PINT87aa+FOXM1 overexpression compared with vector-transfected cells (Figure 6A), as well as in senescent HCC cells compared with proliferating HCC cells (Figure S5B). Besides, PHB2 has been shown to function in mitophagy and cancer 23, 24. Mitochondrial membrane potential (MMP) was significantly increased under PINT87aa+FOXM1 overexpression compared to PINT87aa overexpression alone (Figure 6B). In parallel, we observed that GFP-LC3B spots showed a reduced overlapping with mitochondria, as indicated by Mito-Tracker Red, in the PINT87aa-overexpressing cells, but the two overlapped considerably in the PINT87aa+FOXM1-overexpressing cells (Figure 6C). The proportion of LAMP1 showing lysosomes overlapping with mitochondria indicated by COXIV was higher in the PINT87aa+FOXM1-overexpressing cells than those overexpressing PINT87aa alone (Figure 6D). We further evaluated PINK1 and PARK2 (Parkin) (two specific effectors of mitophagy) levels in HCC cells. The results indicated that the expression of P62, LC3B-II, PINK1, and Parkin was lower in PINT87aa-overexpressing cells but higher in cells with PINT87aa+FOXM1 overexpression (Figure S6). These results indicated that PINT87aa could decrease PHB2 mediated mitophagy.

**Figure 7:**
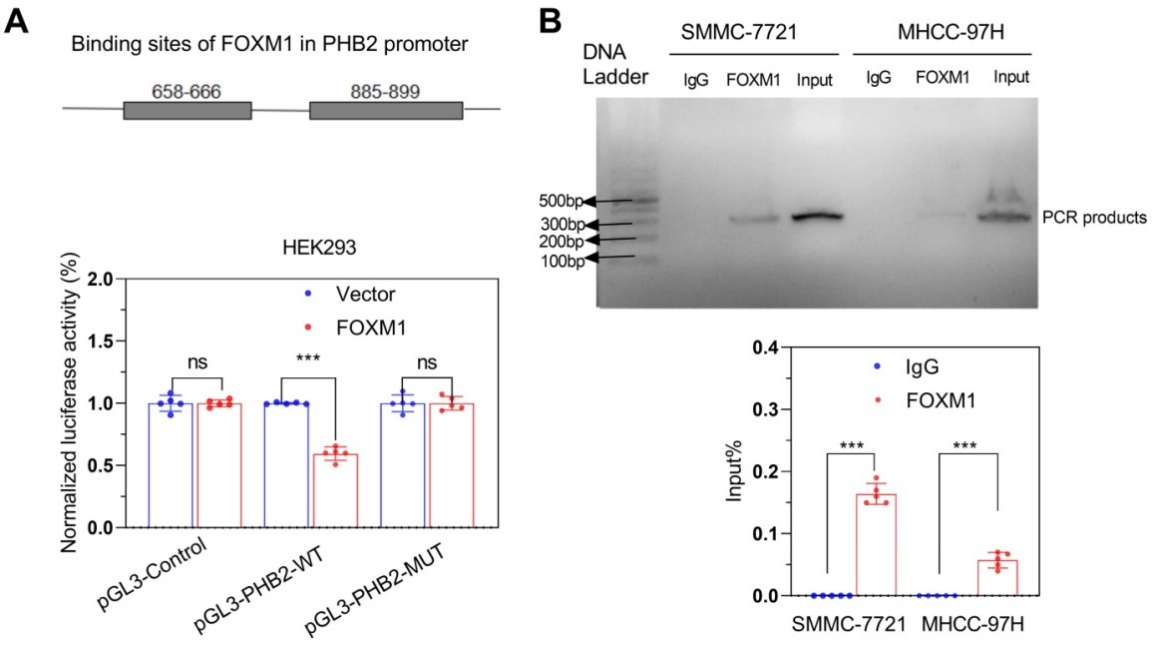
** FOXM1 promoted the transcription of *PHB2*.** (A) Dual-luciferase reporter gene assay and (B) CHIP-PCR verified the transcription of *PHB2* by FOXM1. ***P < 0.001; ns *P* > 0.05.

To validate that FOXM1 could bind to the promoter region of *PHB2*, we first predicted the binding sites of FOXM1 in the *PHB2* promoter using the hTF target database. We observed that 658-666 and 885-899 regions had higher scores, indicating a greater binding possibility. Thus, we constructed dual-site simultaneous mutation and wild-type promoter vectors. Dual-luciferase reporter gene assay showed that the luciferase activity in cells with the mutant vector was higher than with the wild-type promoter vector (Figure 7A); these results were confirmed by the ChIP-PCR assay (Figure 7B).

### Restorative effect of FOXM1 in PINT87aa-overexpressing HCC *in vivo*

**Figure 8:**
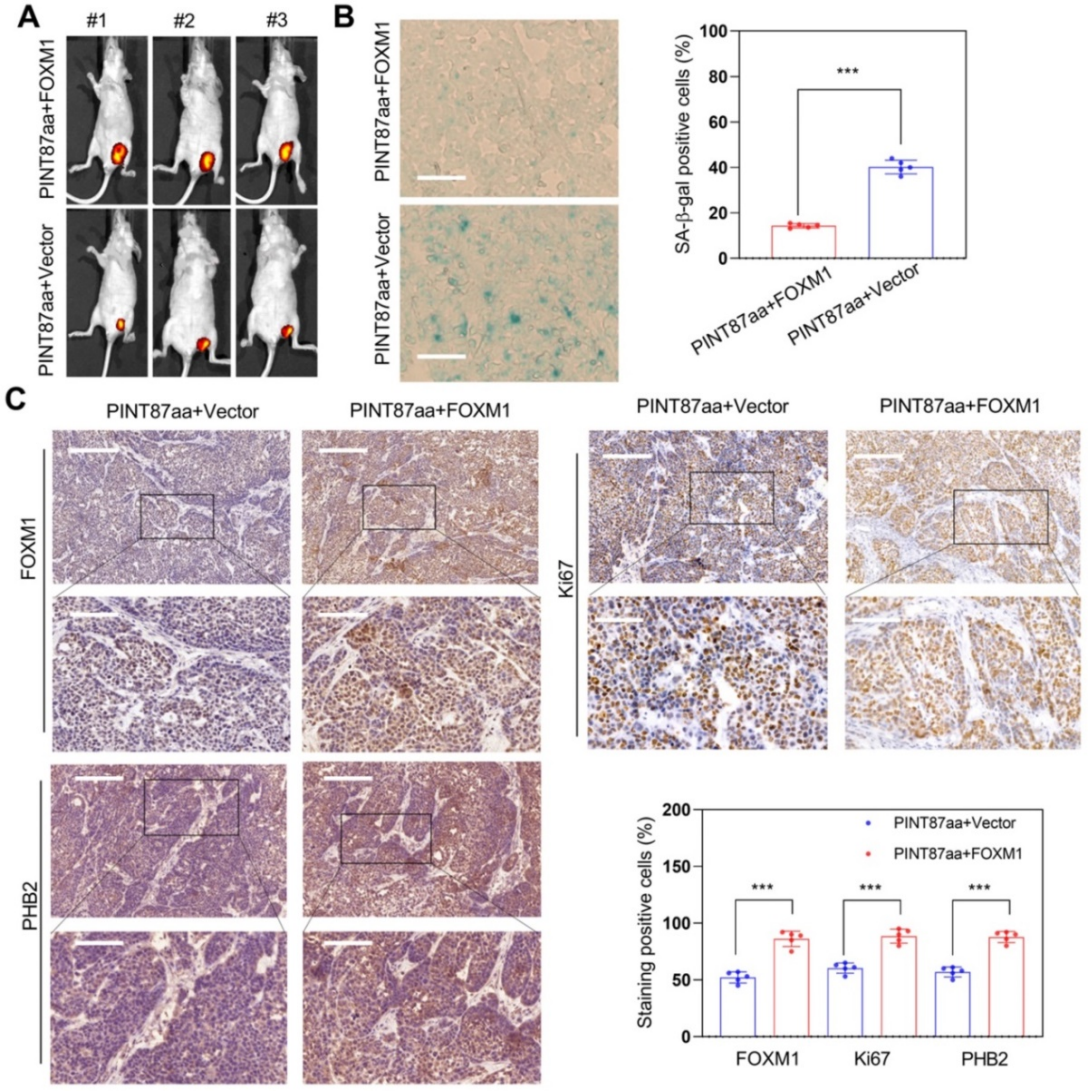
** Restoration effect of FOXM1 on PINT87aa-overexpressing HCC *in vivo*.** (A) Effects of overexpressed FOXM1 on tumor growth by tumorigenicity assay. (B) SA-β-gal staining in implanted tumor tissues with different FOXM1 expression levels (Magnification is ×200). (C) IHC staining of FOXM1, PHB2, and Ki67 in implanted tumors with different FOXM1 expression levels. The scale bar represents 100μm (top) and 50 μm (bottom). ***P < 0.001.

We monitored tumor growth to explore whether FOXM1 could reverse the effect of PINT87aa overexpression in HCC *in vivo* and found a larger tumor volume in the FOXM1 overexpression group (Figure 8A). Next, we observed that HCC tissues with high FOXM1 expression had a lower cellular senescence ratio (Figure 8B). IHC results confirmed that tumors transfected with FOXM1 vectors had higher FOXM1 levels. Besides, Ki67 and PHB2 were higher in the FOXM1 overexpression group (Figure 8C), suggesting that FOXM1 could reverse the PINT87aa-mediated proliferation arrest and cellular senescence.

### Fragment 1 consisting of amino acids from 2 to 39 was required for PINT87aa function

**Figure 9:**
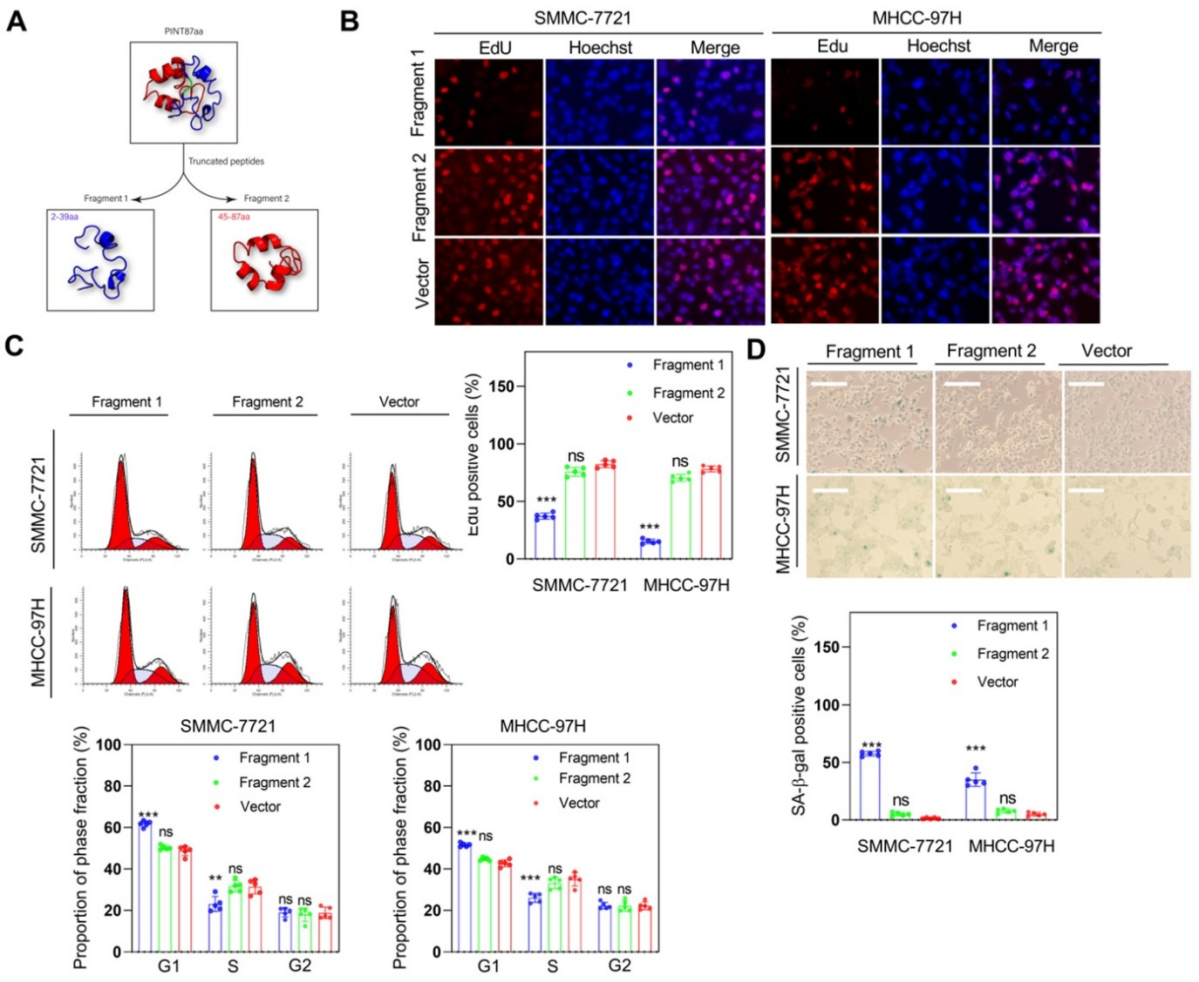
** Fragment 1 containing amino acids 2 to 39 inhibited HCC cell proliferation and induced cellular senescence *in vitro*.** (A) PINT87aa was split into two segments: fragment 1 (2-39 aa) and fragment 2 (45-87 aa). (B-D) Effects of fragment 1 and fragment 2 on cell viability via (B) EdU assay (Magnification is ×100), (C) cell cycle, and (D) cellular senescence (Magnification is ×100) **P < 0.01; ***P < 0.001; ns *P* > 0.05.

**Figure 10:**
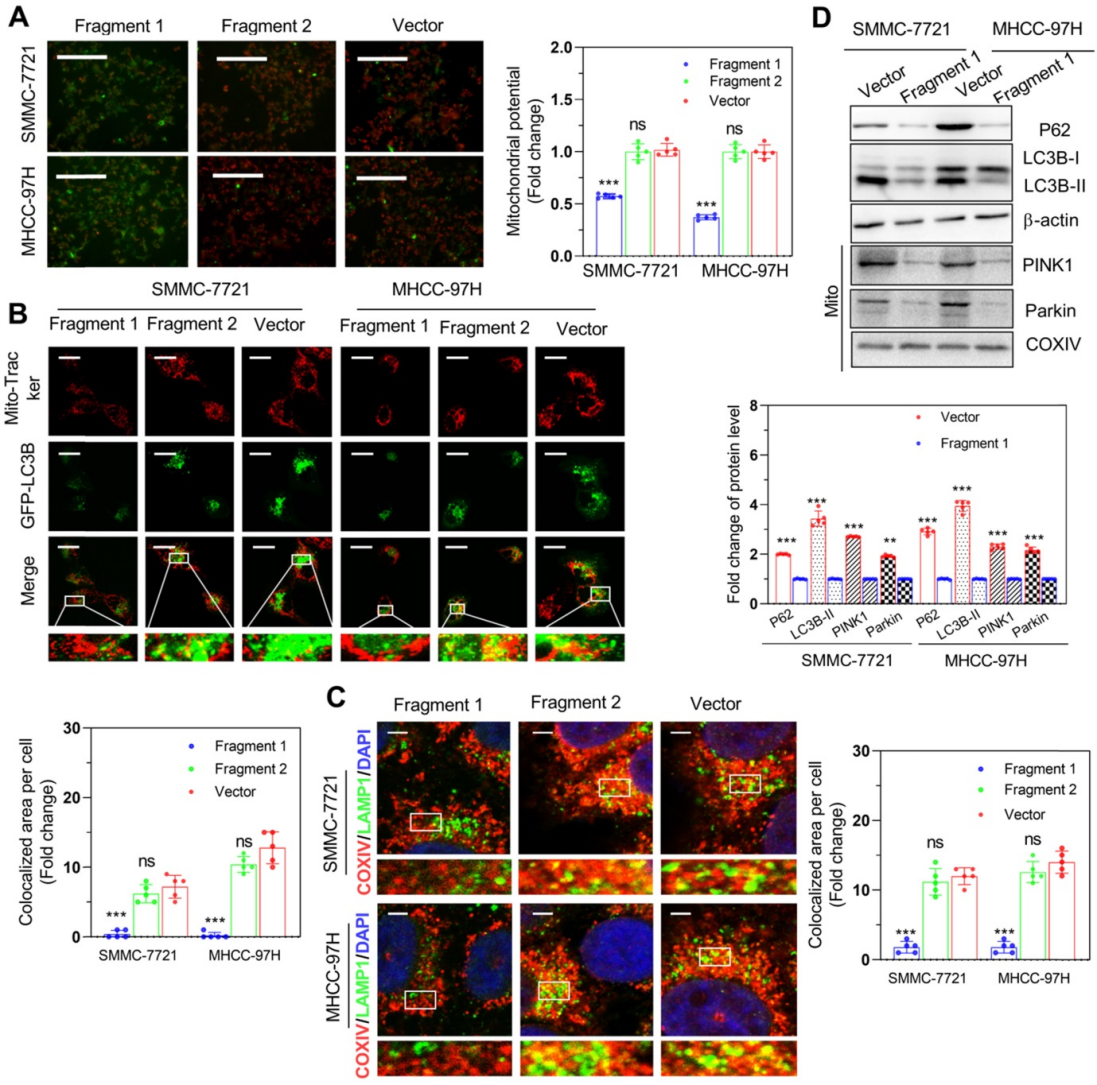
** Fragment 1 containing amino acids 2 to 39 decreased mitophagy *in vitro*.** (A) The MMP (Magnification×100), (B) proportion of GFP-LC3B spots overlapping with the mitochondria by MitoTracker Red (Scale bars = 20 μm), (C) proportion of LAMP1 indicating lysosome spots overlapping with the mitochondria indicated by COXIV (Scale bars = 5 μm), and (D) expression of mitophagy-related proteins: P62, LC3B-II, PINK1, and Parkin detected in SMMC-7721 and MHCC-97H cells with different fragment 1 and fragment 2 levels. **P < 0.01; ***P < 0.001; ns *P* > 0.05.

As previously described, we identified the 30-36 amino acid region was necessary for binding to FOXM1in PINT87aa by using the structural analysis. We divided PINT87aa into two fragments, with fragment 1 including amino acids 2-39 and fragment 2 including amino acids 45-87 (Figure 9A). The two fragments were added to HCC cells at a concentration of 500 uM. The results indicated that fragment 1 could inhibit cell proliferation (Figure 9B), arrest cell cycle (Figure 9C), and increase the proportion of senescent HCC cells (Figure 9D), consistent with the observations from the CellEvent Senescence Green Flow Cytometry Assay (Figure S3C) while fragment 2 had no such effects (Figure 9B-D). Further, fragment 1 could decrease the MMP (Figure 10A) and reduce the overlap of GFP-LC3B spots and mitochondria, as indicated by Mito-Tracker Red staining (Figure 10B). The proportion of LAMP1 indicating lysosomes overlapping with the mitochondria indicated by COXIV was higher in the cells treated with fragment 2 than fragment 1 (Figure 10C). Further, HCC cells treated with fragment 1 exhibited reduced expression of P62, LC3B-II, PINK1, and Parkin (Figure 10D), while these effects were not observed in HCC cells treated with fragment 2 (Figure 10A-C and Figure S7).

**Figure 11:**
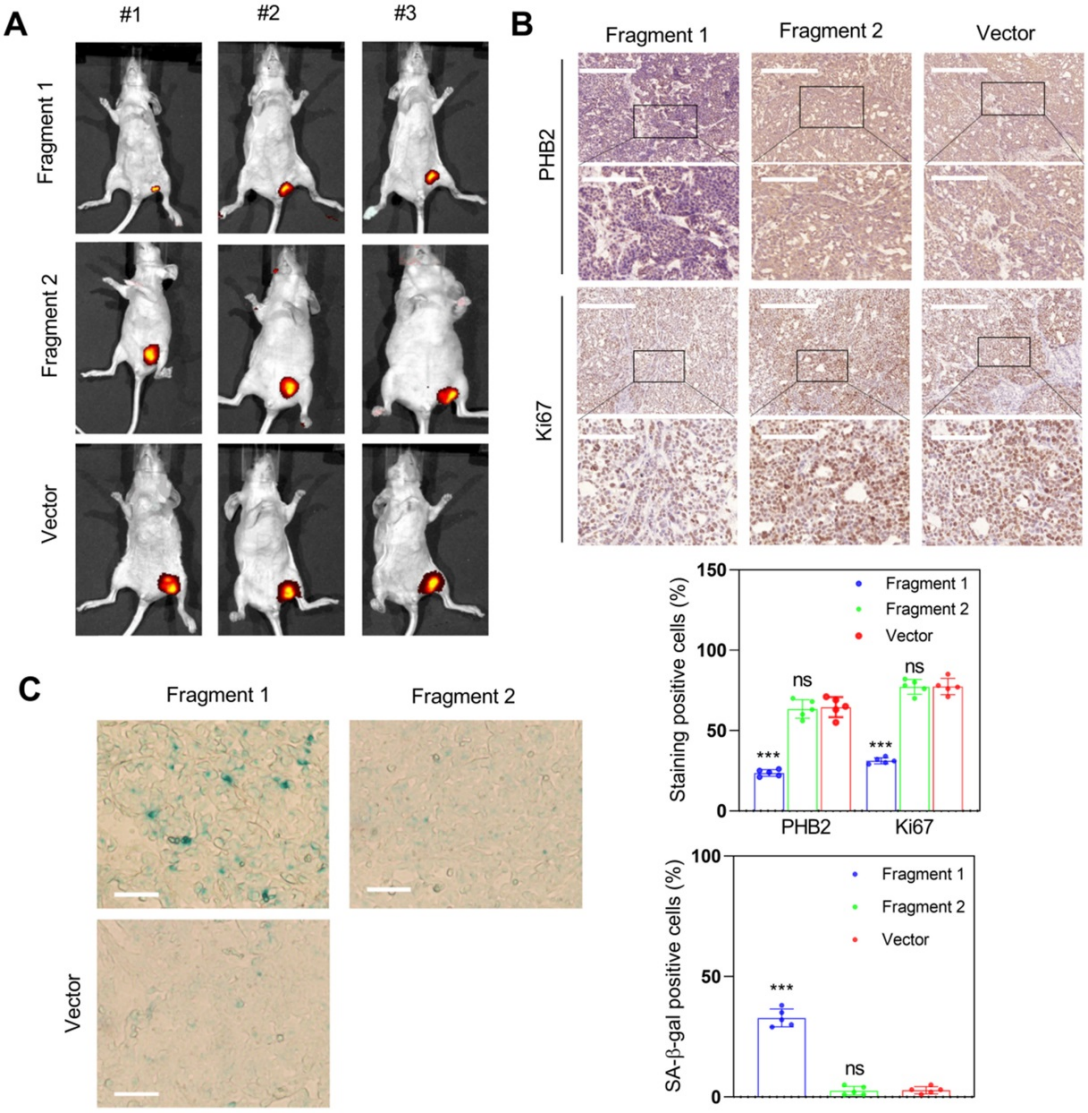
** Fragment 1 with amino acids 2 to 39 was the functional executor of PINT87aa *in vivo*.** (A) Effects of overexpressed fragment 1 and fragment 2 on tumor growth by tumorigenicity assay. (B) IHC staining of PHB2 and Ki67 in implanted tumors with different fragment 1 and fragment 2 levels. The scale bar represents 100μm (top) and 50 μm (bottom). (C) SA-β-gal staining in implanted tumor tissues with different fragment 1 and fragment 2 levels (Magnification, ×200). ***P < 0.001.

To determine whether fragment 1 could function *in vivo*, we constructed fragment 1 and fragment 2 overexpression vectors, transfected them into HCC cells, and inoculated nude mice with these transfected cells to assess tumor formation. The results indicated that overexpression of fragment 1 could significantly inhibit tumor growth (Figure 11A), reduce PHB2 and Ki67 expression (Figure 11B), and increase the proportion of senescent cells in tumor tissues (Figure 11C). We also constructed the truncated PINT87aa-GFP vector lacking 30-36aa and co-transfected with FOXM1 in HEK293 cells. The co-IP results showed no interaction between the truncated PINT87aa-GFP and FOXM1 (Figure S4B), indicating that fragment 1 was the functional FOXM1-binding fragment of PINT87aa.

## Discussion

In this study, we observed overexpression of PINT87aa in senescent HCC cells. Mechanistically, we found that overexpression of PINT87aa reduced cell viability, blocked cell cycle, induced cellular senescence, and inhibited mitophagy by binding to FOXM1 and blocking the transcription of PHB2. Furthermore, we identified that the region containing amino acids 2-39 was required for the PINT87aa function.

Cellular senescence is a stress response to various stimuli that causes genomic perturbations. Specific cellular senescence-related lncRNAs have been described, such as p53-dependent and -independent SENEBLOC, SNHG12, and PANDA 25-27. In the present study, LINC-PINT overexpression was confirmed in an oxidative stress-induced HCC cell model of senescence. Recently, we have found the small open reading frames (sORF) in lncRNAs were reported to have the potential to encode peptides or proteins, and these encoded products, such as FBXW7-185aa and SHPRH-146aa, could play an important role in human cancers 28, 29. In addition, our previous study revealed that *LINC-PINT* could encode a peptide consisting of 87 amino acids (PINT87aa), which exerted a tumor-suppressive effect in gliomas 18. In this study, we found upregulation of PINT87aa in senescent HCC cells suggestive of a potential pro-senescence role, which was confirmed by the observation that PINT87aa overexpression significantly reduced cell viability and induced G1 phase cell cycle arrest. Moreover, PINT87aa was expressed in HCC cells independent of the *TP53* status and could induce the senescence of p53 wild-type and mutant HCC cells, indicating its potential for broader targeting in HCC treatment. We also found that PINT87aa overexpression alone could induce cellular senescence, suggesting that it might act as a driver of cellular senescence.

It has been reported that mitophagy and cellular senescence are interdependent, and their imbalance is involved in certain diseases 30. For example, restoration of mitophagy could reverse senescence and restore the stemness of satellite cells 31, while senescence regulator p53 could inhibit mitophagy 32. Our data revealed that dysfunctional mitochondria were maintained in senescent cells by inhibiting mitophagy while eliminated from proliferating cells by mitophagy, which was beneficial to cell proliferation. Furthermore, we performed rescue experiments to verify the FOXM1 function in PINT87aa-mediated cell cycle arrest, cellular senescence, and mitophagy inhibition and found that FOXM1 was involved in the regulation of mitochondrial function. For example, PRX3 induced by FOXM1 could maintain the mitochondrial function of colon cancer cells 33. We confirmed that FOXM1 could promote the transcription of *PHB2* and a negative regulatory effect of PINT87aa on PHB2 expression. These results were consistent with a previous report describing that PHB2 promoted LC3-mediated mitophagy through the PINK1-PRKN/Parkin axis 23, 24. However, the detailed molecular mechanism linking mitophagy and senescence has not been fully elucidated.

Peptide/small protein drugs, such as insulin and mifamurtide, used to treat human diseases 34, 35, have high specificity and less cytotoxicity to normal tissues. In this context, peptides/small proteins encoded by lncRNAs have good prospects for tumor therapy application, as exemplified by the HOXB-AS3 peptide 36. Herein, we found that PINT87aa, the peptide encoded by LINC-PINT exon 2, could induce cell cycle arrest and cellular senescence. We further identified a fragment consisting of 2-39 amino acids responsible for the effects of PINT87aa, suggesting the PINT87aa fragment as a promising anti-tumor drug candidate.

Nevertheless, this study has some limitations. We studied the role of PINT87aa in pro-senescence therapy and its effects on the mitophagy of HCC cells. HCC is a malignant disease with extensive molecular heterogeneity. Therefore, in the future, it would be worth exploring other potential regulatory mechanisms of PINT87aa in other HCC sub-types. Also, investigating PINT87aa upstream regulation would help clarify the comprehensive tumor-suppressive roles of PINT87aa.

In summary, we observed that PINT87aa was overexpressed in senescent HCC cells. Importantly, we demonstrated that PINT87aa could induce cell cycle arrest and cellular senescence by directly binding to FOXM1 to block *PHB2* transcription. This is the first study reporting the anti-proliferation and pro-senescence activities of PINT87aa in HCC cells and providing a rationale for exploring PINT87aa as an anti-cancer drug for HCC.

## Supplementary Material

Supplementary methods, figures and tables.

## Figures and Tables

**Table 1:** Fold-change of FOXM1 and its downstream genes involved in cell cycle or proliferation in HCC cells with overexpression of PINT87aa.

Gene symbol	Average fold-change (PINT87aa/Vector)	*P-*Value
ATM	0.070249911	<0.001
BMI1	0.240388321	<0.01
CCND1	0.649384139	<0.05
CCNE1	0.610136679	<0.05
CDK2	0.359107748	<0.001
CDK4	0.576862385	<0.01
CDK6	0.620572263	<0.001
CDKN1A	1.530619199	<0.001
CHEK1	0.206240548	<0.001
CHEK2	0.1932231	<0.001
E2F1	0.857284592	<0.05
E2F3	0.403138224	<0.001
ETS1	0.325011416	<0.001
ETS2	0.803896360	<0.01
RB1	0.412061260	<0.01
CDKN2A	1.392837752	<0.05
MDM2	0.516847083	<0.001
RBL2	1.05180420	>0.05
TWIST1	1.25444096	<0.05
FOXM1	0.98369748	>0.05

ATM: ATM serine/threonine kinase; BMI1: BMI1 proto-oncogene, polycomb ring finger; CCND1: cyclin D1; CCNE1: cyclin E1; CDK2: cyclin-dependent kinase 2; CDK4: cyclin-dependent kinase 4; CDK6: cyclin-dependent kinase 6; CDKN1A: cyclin-dependent kinase inhibitor 1A; CDKN2A: cyclin-dependent kinase inhibitor 2A; CHEK1: checkpoint kinase 1; CHEK2: checkpoint kinase 2; E2F1: E2F transcription factor 1; E2F3: E2F transcription factor 3; ETS1: ETS proto-oncogene 1, transcription factor; ETS2: ETS proto-oncogene 2, transcription factor; RB1: RB transcriptional corepressor 1; RBL2: RB transcriptional corepressor-like 2; MDM2: MDM2 proto-oncogene; TWIST1: twist family bHLH transcription factor 1. FOXM1: forkhead box M1; HCC: hepatocellular carcinoma.

## Abbreviations

HCC: hepatocellular carcinoma
LINC-PINT: long intergenic non-protein coding RNA, p53-induced transcript
PHB2: prohibitin 2
CHIP-PCR: Chromatin immunoprecipitation-polymerase chain reaction
DDR: DNA damage response
lncRNA: long non-coding RNA
FISH: fluorescence *in situ* hybridization
FOXM1: forkhead box M1
IF: immunofluorescence
IHC: immunohistochemistry
CO-IP: Co-immunoprecipitation
circPINT: circular LINC-PINT
linePINT: linear LINC-PINT
RS: replicative senescence
OIS: oncogene-induced senescence
MMP: mitochondrial membrane potential
SA-lncRNAs: senescence-associated lncRNAs
SA-β-Gal: senescence-associated β-galactosidase
LC3B: light chain 3 beta
SPHC: senescent primary hepatocytes
LAMP1: lysosomal associated membrane protein 1
COXIV: cytochrome c oxidase subunit 4I1
