# Supplementary material for: Cellular senescence in hepatocellular carcinoma induced by a long non-coding RNA-encoded peptide PINT87aa by blocking FOXM1-mediated PHB2
Source: Theranostics. 2021 Mar 4;11(10):4929–44. doi: 10.7150/thno.55672 (PMC7978318; doi:10.7150/thno.55672)
Supplement: Supplementary file 1 — Supplementary methods, figures and tables. [file thnov11p4929s1.pdf]

Supplementary Methods

Identification of Senescence-associated lncRNAs from in silico models

The microarray data of senescence cells which performed on the Affymetrix Human Genome U133 Plus 2.0 Array platform were downloaded from the Gene Expression Omnibus (GEO, http://www.ncbi.nlm.nih.gov/geo). To generate comparable data and avoid species variation, we only included the datasets that employed human IMR-90 cell line, a most widely used senescent cell model. Finally, five datasets, including GSE19018 and GSE36640 (to generate the RS model) and GSE19864, GSE40349 and GSE60652 (to generate the OIS model) were used in this study. The raw CEL based files were downloaded and normalized by Robust Multi-array Average (RMA) method. LncRNA annotation from microarray data was conducted according to our previous study [1]. The differentially expressed lncRNAs, which were identified as fold change ≥2.0 and t-test P value <0.01, in replicative senescence (RS) and oncogene-induced senescence (OIS) models, respectively. By overlapping the two lists, common differentially expressed lncRNAs were finally identified as senescence-associated lncRNAs.

Cellular senescent model conduction

In this study, we treated HCC cells by hydrogen peroxide (H_2O_2), which is a widely used method to induce cellular senescence. The concentration of H2O2 ranged from 0 to 200 μM at interval of 25 μM. SA-β-gall staining, the widely accepted senescent biomarker, was employed to evaluate the senescent rate of HCC cells after H2O2 treatment at 0h, 6h,12h, 24h, 36h and 48h.

Other methods

The binding sites of FOXM1 in *PHB2* promoter regions were predicted by hTFtarget (http://bioinfo.life.hust.edu.cn/hTFtarget#!/prediction). GSE60032 contained FOXM1 binds directly to non-consensus sequences in the human genome was downloaded from the GEO.

References

1. L. Wang, Y. Hu, X. Xiang, K. Qu, Y. Teng, Identification of long non-coding RNA signature for paclitaxel-resistant patients with advanced ovarian cancer, Oncotarget, 8 (2017) 64191-64202.

Supplementary table 1. Primers used in this research.

Gene symbol	Forward primer（5'to 3'）	Reverse primer（5'to 3'）
LINC-PINT	GAAGAGGGACTGGAACCATC	CTCATCTGCGAGGAGACAGG
TMPO-AS1	CTTTTGTGCGCCGTTTCCT	CCCAGAGACGAAAGCTGCTT
LOC100129034	CAGAACGGCGGCAATAATGT	TGGGTCGGGAACTTTGGGTA
LINC00973	TTGAAGGCTTCCTGGTCTGAG	AGGCTTACATTCCAGCTGTGT
LOC101928198	TGCTCTAGCAAAGCCTCACTT	GGGAGAATGAGATGCTGGCTT
LOC101930593	TCCTTTGGACTGTGCGCTT	TGTAATTTCTGGGCTCCCTCC
PHB2-Promoter	TCCAGCCTGGTAGAGACTGAG	CTCTCTAATATCCACCTATGA
β-actin	GGCGGCACCACCATGTACCCT	AGGGGCCGGACTCGTCATACT
CircPINT	GCGTTCAGCCCTGGGGTCATAT	CAGTTTTTCTCTTCCGCAGCTA
LinePINT	GGCTTGGCTAGTTGGAGAGTTAC	AACTGAAACCAGACCTAAGGTTTTG

ATM	TTTTCAACCAGTTTTCCGTTACTTC	ACACTGCGCGTATAAGCCAAT
BMI1	TGATGTGTGTGCTTTGTGGAGG	GTGGTCTGGTCTTGTGAACTTGG
CCND1	AAAGGAAGCAAGAACCCAT	GTCCGAGATTATCATTACCC
CCNE1	TACACCAGCCACCTCCAGACAC	CCTCCACAGCTTCAAGCTTTTG
CDK2	CCAGGAGTTACTTCTATGCCTGA	TTCATCCAGGGGAGGTACAAC
CDK4	CGGAGTGAGCAATGGAGTG	CTAAGGGTAAATCAGGGATAGGG
CDK6	GCACATCAGTAATTCAGTAGAC	ATAGCCAGGAGAGTAATTCATC
CDKN1A	AGTCAGTTCCTTGTGGAGCC	CATTAGCGCATCACAGTCGC
CHEK1	CAGAATTTCAACCTTCGGTGTG	TCTTCACTGCGACTGCTTCTTC
CHEK2	TGTCCCTCCCAAACCAGTAGTTGT	TTCACAGCCCCATGGCAGCG
E2F1	AGTCCCAGCCAGTCTCTACTCA	TGCCCATCCGGGACAA
E2F3	TGACCCAATGGTAGGCACAT	CATCTAGGACCACACCGACA
ETS1	TTACTCAGCGCCTCGTCCT	GATCCCCAGTCGTTGCTGTT
ETS2	CACGGGCCTAATCCTCAGTC	GAAGGTTTTGTAATTTGGCC
RB1	TTTGTAACGGGAGTCGGGAGA	CTCAAGCCTGACGAGAGGCAG
CDKN2A	TGGTCACTGTGAGGATTCAGC	TCGCACGAACTTCACCAAGA
MDM2	GCTCATCCTTTACACCAACTCC	TACCTCCCTTATAGACCATTCACG
RBL2	GGAGGAAATTGGGACTCTCTCA	AGACGACTCAAGCTATGCGTA
TWIST	GTCCGCAGTCTTACGAGGAG	GCTTGAGGGTCTGAATCTTGCT
FOXM1	TGCAGCTAGGGATGTGAATCTTC	GGAGCCCAGTCCATCAGAACT

ATM: ATM serine/threonine kinase; BMI1: BMI1 proto-oncogene, polycomb ring finger; CCND1: cyclin D1; CCNE1: cyclin E1; CDK2: cyclin dependent kinase 2; CDK4: cyclin dependent kinase 4; CDK6: cyclin dependent kinase 6; CDKN1A: cyclin dependent kinase inhibitor 1A; CDKN2A: cyclin dependent kinase inhibitor 2A; CHEK1: checkpoint kinase 1; CHEK2: checkpoint kinase 2; E2F1: E2F transcription factor 1; E2F3: E2F transcription factor 3; ETS1: ETS proto-oncogene 1, transcription factor; ETS2: ETS proto-oncogene 2, transcription factor; RB1: RB transcriptional corepressor 1; RBL2: RB transcriptional corepressor like 2; MDM2: MDM2 proto-oncogene; TWIST1: twist family bHLH transcription factor 1. FOXM1: forkhead box M1.

Supplementary table 2. Antibodies used in this research.

Name	Source	Catalog Number	Application and dilution
PINT87aa	Rabbit	Genscript, Nanjing	WB: 1:1000; IHC:1:50<br>IP: 1:200; IF:1:500
FOXM1	Rabbit	(D3F2B) #20459, CST	WB: 1:1000; IHC:1:600<br>IP: 1:100
	Mouse	Sc-376471, Santa Cruz Bio	IP: 1:100; IF:1:200

PHB2	Rabbit	CY8226, Abways	WB: 1:1000; IHC:1:200
Ki67	Rabbit	GB111141, Servicebio	IHC:1:1500
LC3B	Rabbit	(D11) #3868, CST	WB: 1:1000
PINK1	Rabbit	(D8G3) #6946, CST	WB: 1:1000
Parkin	Rabbit	CY6641, Abways	WB: 1:1000
P62	Rabbit	18420-1-AP, Proteintech	WB: 1:1000
β-actin	Mouse	YM3028, Immunoway	WB: 1:5000
COXIV	Rabbit	11242-1-AP, Proteintech	WB: 1:5000; IF:1:200
LAMP1	Mouse	Sc-20011, Santa Cruz Bio	IF:1:200
Goat Anti-Mouse IgG (H+L)-Alexa Fluor 647	Mouse	RS3808, Immunoway	IF: 1:500
Goat Anti-Rabbit IgG (H+L)-Alexa Fluor 488	Rabbit	RS3211, Immunoway	IF: 1:500
Goat Anti-Mouse IgG (H+L)-HRP	Mouse	AB0102, Abways	WB: 1:10000
Goat Anti-Rabbit IgG (H+L)-HRP	Rabbit	AB0101, Abways	WB: 1:10000

Supplementary table 3. Fold change of senescence-associated lncRNAs in HCC cells

Gene symbol	Average fold change (Senescent/Proliferating cells)	*P*-Value
LINC-PINT	10.032	<0.001
TMPO-AS1	0.221	<0.05
LOC100129034	1.245	>0.05
LINC00973	1.197	>0.05
LOC101928198	1.003	>0.05
LOC101930593	0.945	>0.05

Supplementary figures and figure legends

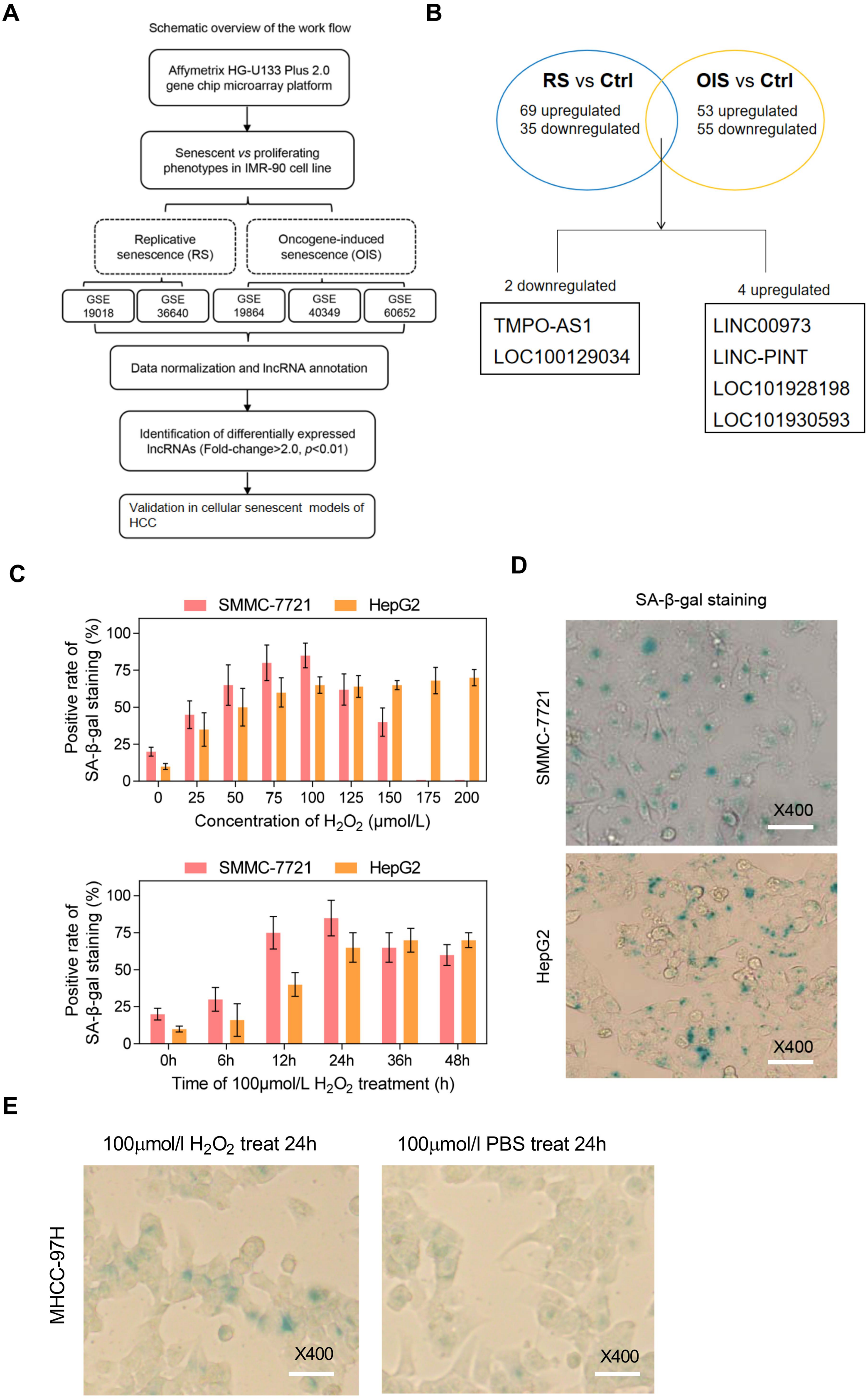

Figure S1. Identification of SA-lncRNAs from *in silico* models and conduction of cellular senescent model. (A) The workflow of identification of SA-lncRNAs in senescnet HCC cells. (B) Schematic diagram of identifying SA-lncRNAs *in silico* models. (C) The dose-dependent and time-dependent manner of H_2O_2 induced cellular senescence. (D) SA-β-gal staining in H_2O_2 induced senescent SMMC-7721 and HepG2 cells. (E) SA-β-gal staining validated the efficiency of H_2O_2 induced senescent model in MHCC-97H cells. H_2O_2, hydrogen peroxide; SA-lncRNAs, senescence-associated lncRNAs.

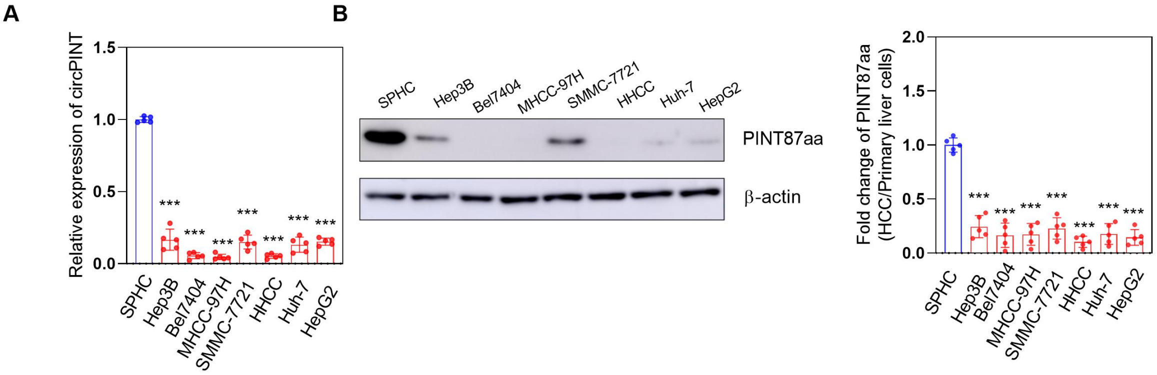

Figure S2. The expression of circPINT and PINT87aa in senescent primary liver cells and HCC cell lines. The expression of circPINT(A) and PINT87aa (B) in senescent primary hepatocytes (SPHC) and HCC cell lines. ***$P < 0.001$. SPHC: Senescent primary hepatocytes.

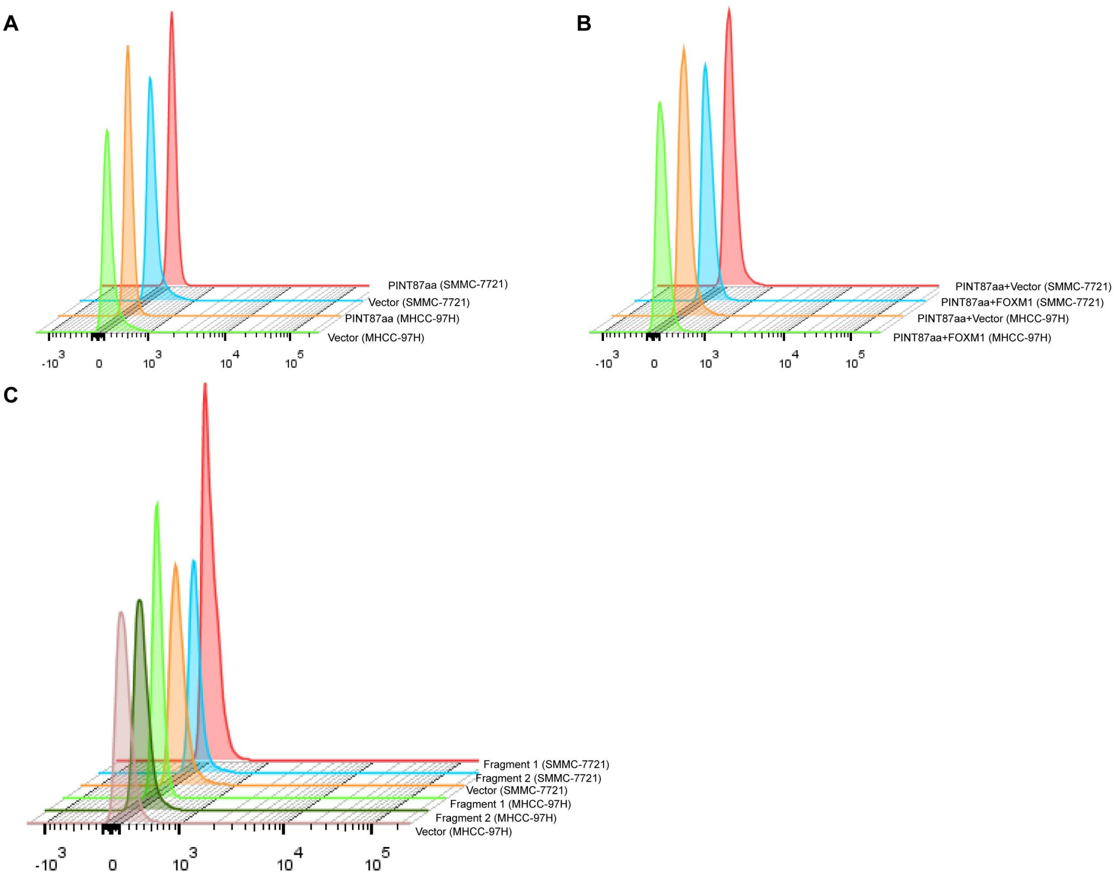

Figure S3. CellEvent Senescence Green Flow Cytometry Assay validated PINT87aa and

fragment 1 induced cellular senescence and reverse effect of FOXM1 on PINT87aa.

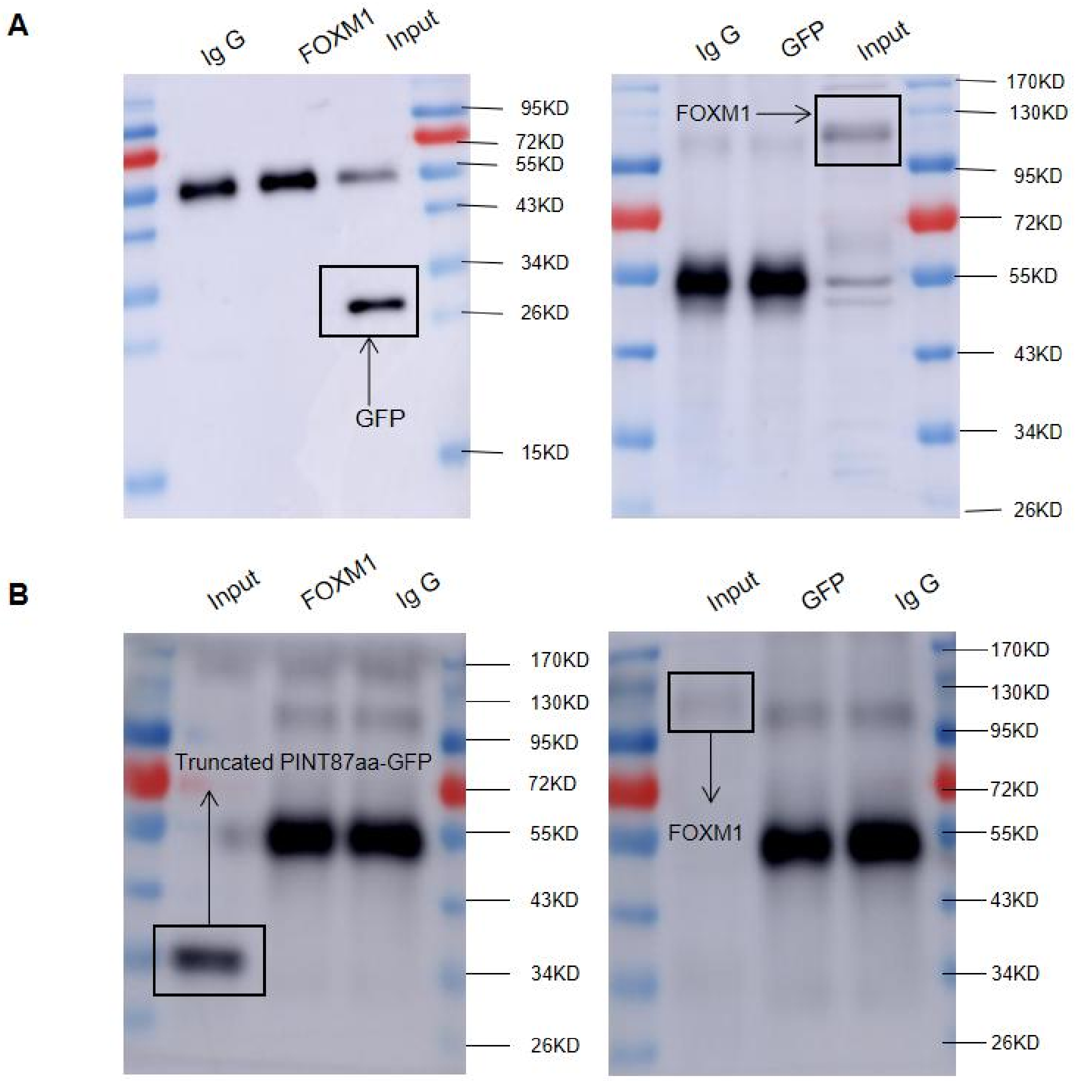

Figure S4. Co-immunoprecipitation verified the combination ability of FOXM1 and GFP or truncated PINT87aa-GFP. (A) Co- immunoprecipitation was performed using an anti-GFP antibody and anti-FOXM1 antibody in FOXM1 and GFP co-transfected HEK293 cells respectively. (B) Co- immunoprecipitation was performed using an anti-GFP antibody and anti-FOXM1 antibody in truncated PINT87aa-GFP and FOXM1 co-transfected HEK293 cells respectively.

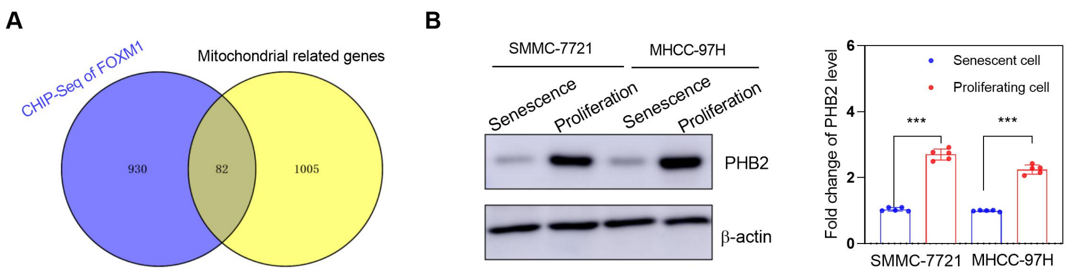

Figure S5. Mitophagy was inhibited in senescent HCC cells. (A) The overlapping of FOXM1 target genes of the GEO dataset GSE60032 and genes related to mitochondrial

function. (B) The expression of PHB2 was detected in senescent and proliferating HCC cells. ***$P < 0.001$.

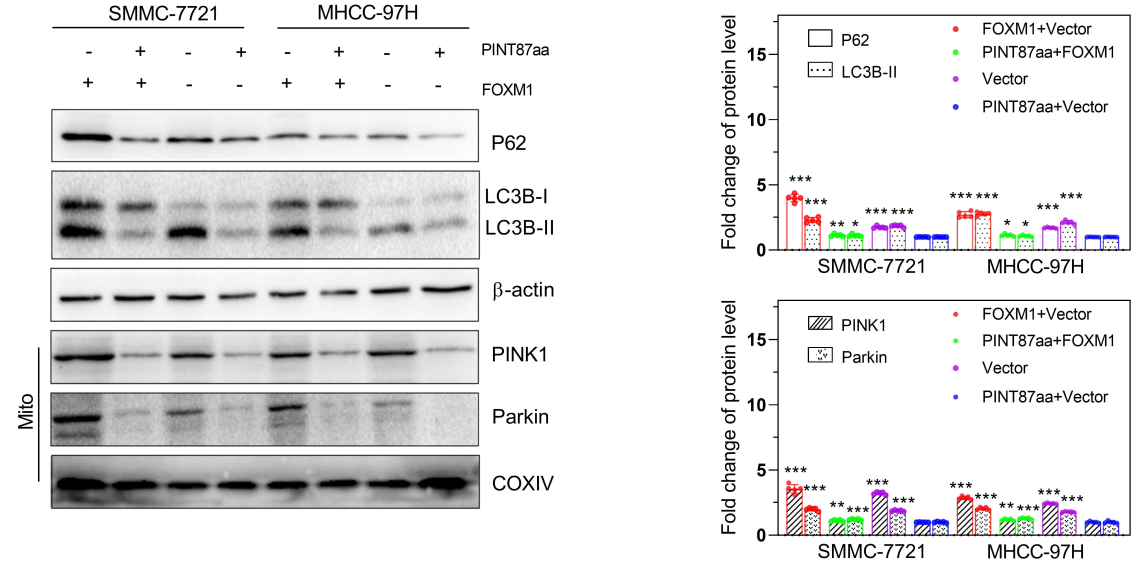

Figure S6. The reverse effects of FOXM1 on the expression of mitophagy-related protein. *$P < 0.05$; **$P < 0.01$; ***$P < 0.001$.

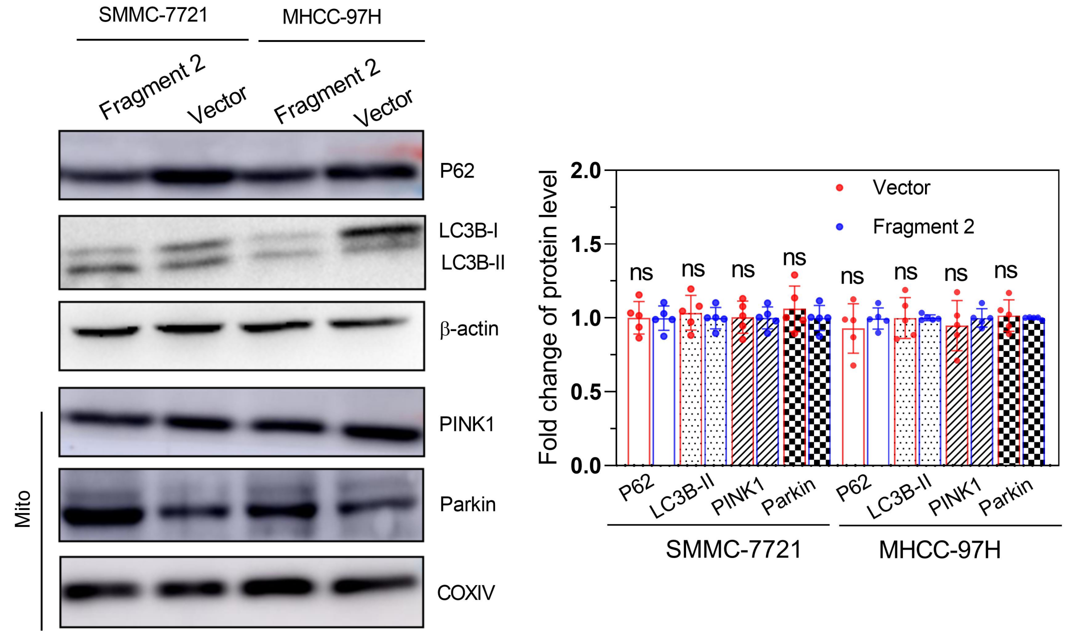

Figure S7. The effects of fragment 2 on the expression of mitophagy-related protein. ns $P > 0.05$.

Figure 6C:

C

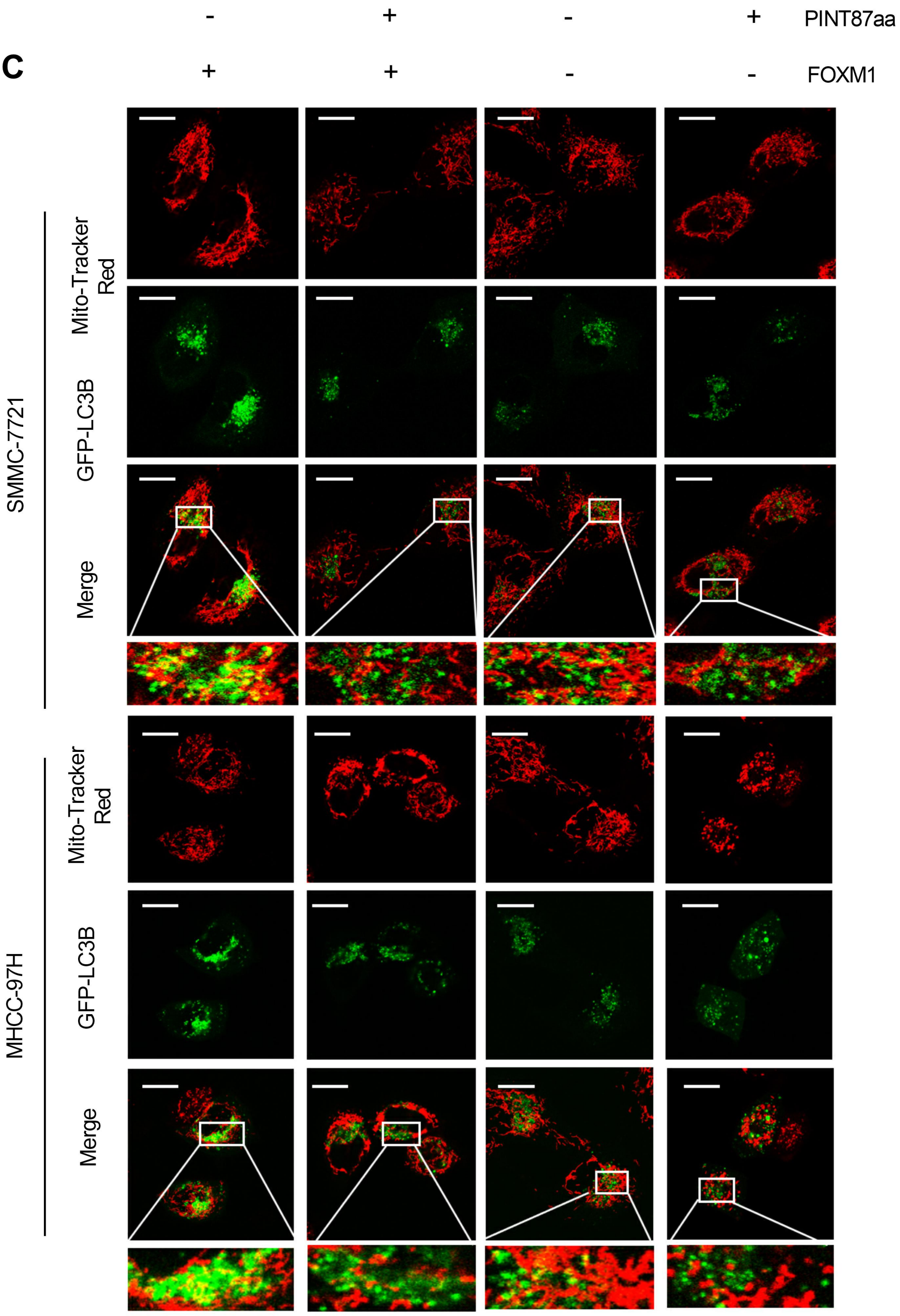

-
+
-
+
PINT87aa
+
+
-
-
FOXM1
SMMC-7721
Mito-Tracker Red
GFP-LC3B
Merge
MHCC-97H
Mito-Tracker Red
GFP-LC3B
Merge
